# MeDIP combined with in-solution targeted enrichment followed by NGS: Inter-individual methylation variability of fetal-specific biomarkers and their implementation in a proof of concept study for NIPT

**DOI:** 10.1371/journal.pone.0199010

**Published:** 2018-06-11

**Authors:** Anna Keravnou, Marios Ioannides, Charalambos Loizides, Kyriakos Tsangaras, Achilleas Achilleos, Petros Mina, Elena Kypri, Michael D. Hadjidaniel, Maria Neofytou, Skevi Kyriacou, Carolina Sismani, George Koumbaris, Philippos C. Patsalis

**Affiliations:** 1 Translational Genetics Team, The Cyprus Institute of Neurology and Genetics, Nicosia, Cyprus; 2 NIPD Genetics Ltd., Nicosia, Cyprus; 3 Department of Cytogenetics and Genomics, The Cyprus Institute of Neurology and Genetics, Nicosia, Cyprus; Tel Aviv University, ISRAEL

## Abstract

DNA methylation is the most characterized epigenetic process exhibiting stochastic variation across different tissues and individuals. In non-invasive prenatal testing (NIPT) fetal specific methylated regions can potentially be used as biomarkers for the accurate detection of fetal aneuploidies. The aim of this study was the investigation of inter-individual methylation variability of previously reported fetal-specific markers and their implementation towards the development of a novel NIPT assay for the detection of trisomies 13, 18, and 21. Methylated DNA Immunoprecipitation (MeDIP) combined with in-solution targeted enrichment followed by NGS was performed in 29 CVS and 27 female plasma samples to assess inter-individual methylation variability of 331 fetal-specific differentially methylated regions (DMRs). The same approach was implemented for the NIPT of trisomies 13, 18 and 21 using spiked-in (n = 6) and pregnancy samples (n = 44), including one trisomy 13, one trisomy 18 and four trisomy 21. Despite the variability of DMRs, CVS samples showed statistically significant hypermethylation (p<2e-16) compared to plasma samples. Importantly, our assay correctly classified all euploid and aneuploid cases without any false positive results (n = 44). This work provides the starting point for the development of a NIPT assay based on a robust set of fetal specific biomarkers for the detection of fetal aneuploidies. Furthermore, the assay’s targeted nature significantly reduces the analysis cost per sample while providing high read depth at regions of interest increasing significantly its accuracy.

## Introduction

The current gold standard in prenatal diagnosis involves invasive testing of fetal DNA through amniocentesis and chorionic villus sampling (CVS). These procedures are associated with a considerable risk of spontaneous abortion, estimated at 0.1–0.2% [1]. The identification of cell free fetal DNA (cffDNA) in maternal circulation in 1997 [2], has greatly facilitated the development of non-invasive prenatal testing (NIPT) that could be offered to all pregnant women without any risk of miscarriage [3].

In the last decade several approaches have been developed for NIPT of fetal aneuploidies, including DNA-based approaches, investigation of targeted-fetal specific mRNAs [4] or fetal-specific proteins [5]. Early studies for NIPT of trisomy 21 were focused on the detection and quantification of paternally-inherited loci using SNP arrays or Real-time quantitave PCR [6,7]. However, the limited amount of cffDNA in the presence of an excess maternal DNA and the limited number of fetal-specific markers presented a challenge for the development of NIPT applications [6].

The advent of next generation sequencing (NGS) has greatly facilitated the development of NIPT. Initial efforts using massive parallel sequencing (MPS) showed high potential in the non-invasive detection of fetal aneuploidies [8,9]. More recently, targeted sequencing approaches in which selected cffDNA sequences are used provided more efficient, accurate and cost effective NIPT methods [10,11,12,25]. Epigenetic-based approaches have also gained ground in recent years for the identification of fetal aneuploidies utilizing methylation based assays [13–16]. Towards the identification of fetal-specific biomarkers for NIPT, much interest has been focused on the methylation differences between maternal and fetal DNA by employing a variety of methods including sodium bisulfite conversion and methylation-sensitive restriction digestion yielding a small number of fetal specific differentially methylated regions (DMRs) [17–19].

Using MeDIP coupled with high resolution array-CGH, our group identified more than 2000 DMRs on chromosomes 21, 18, 13, X and Y [20,21]. This led to the development of a proof of concept NIPT assay based on MeDIP coupled with quantitative PCR (qPCR) for the detection of Down syndrome, resulting in high sensitivity and specificity [13,14]. Additionally, we identified and confirmed 331 genome-wide fetal-specific DMRs by combining for the first time MeDIP and in-solution hybridization followed by NGS [16]. The current study broadens the findings of our previous work by investigating the inter-individual methylation variability of the 331 fetal-specific DMRs using multiple fetal and maternal samples [16]. Furthermore, we present a novel approach for non-invasive detection of trisomies 13, 18 and 21 based on these validated DMRs by utilizing MeDIP coupled with in-solution targeted enrichment followed by NGS. Despite the presence of inter-individual methylation variability, this work confirms the distinct methylation pattern of the previously selected DMRs thus setting the foundation for the development of a novel NIPT assay for the detection of fetal trisomies 13, 18 and 21.

## Materials and methods

### Sample collection and DNA extraction

The study was approved by the Cyprus National Bioethics Committee and informed written consent was obtained from all participants. For the investigation and characterization of inter-individual methylation variability of selected DMRs, 29 first trimester (11–14 weeks of gestation) CVS (24 euploid and five trisomy 21) and 27 female non-pregnant plasma samples were used. All CVS underwent karyotyping and/or Quantitative-Fluorescent PCR (QF-PCR) analysis to confirm their status. Non-pregnant female plasma samples were obtained from Sera Laboratories International Ltd (Sussex, UK).

For the development of the NIPT assay, 44 peripheral blood samples were collected into two 8 mL EDTA-containing tubes from women with singleton pregnancies (11–14 weeks of gestation). A mean of 8 ml plasma was isolated via a double centrifugation protocol, as previously described [22]. All pregnancy samples were obtained from collaborating centers of the Translational Genetics Team and the Department of Cytogenetics and Genomics at the Cyprus Institute of Neurology and Genetics (Nicosia, Cyprus). Among the 44 cases, six were aneuploid, including one trisomy 13, one trisomy 18 and four trisomy 21. Aneuploid cases were confirmed by karyotyping.

Genomic DNA was extracted from CVS samples using the QIAamp Mini kit (Qiagen, Hilden, Germany) according to the manufacturer’s instructions. Cell-free DNA was extracted using the QIAsymphony DSP virus/pathogen midi kit (Qiagen) on QIAsymphony SP/AS.

Total cfDNA concentration and fetal fraction were estimated using Taqman probes, targeting the DYS14 and β-globin loci as previously described [23], or an in-house assay based on methylation-sensitive restriction digestion followed by a multiplex Taqman droplet digital PCR (ddPCR) quantification (manuscript in preparation).

### Preparation of synthetic pregnancy samples

Towards the development of a NIPT assay, our initial efforts were focused on the implementation of MeDIP combined with in-solution targeted enrichment on synthetic affected and non-affected plasma samples. DNA obtained from plasma of non-pregnant female samples were spiked-in with DNA obtained from euploid or trisomy 21 male CVS samples at concentrations of 5%, 10% and 20%. Before mixing, fetal DNA was sheared to an average size of 230bp using the Bioruptor Twin Sonicator (UCD-400, Diagenode, Liege, Belgium). The concentration of spiked-in DNA into plasma DNA was measured using qPCR using the DYS14 and β-globin loci as previously described [23].

### Sequencing-library preparation and methylated DNA Immunoprecipitation (MeDIP)

DNA from CVS and non-pregnant female plasma were used to generate libraries using standard preparation methods. Genomic DNA obtained from CVS samples ranging from 12–30 ng was sheared to an average size of 230bp using the Bioruptor Twin Sonicator (UCD400, Diagenode, Liege, Belgium) and run on the TapeStation 2200 (Agilent Technologies, Santa Clara, CA USA) for fragment size verification. Blunt-ending and sequencing-adaptor ligation were performed prior to MeDIP using NEB Blunting and Ligase enzymes (NEB, Ipswich, UK) as previously described [16,24,25]. For synthetic pregnancy samples and pregnancy cases, library preparation was performed using the iDEAL Library Preparation kit (Diagenode) following the manufacturer’s protocol. MeDIP was performed as described previously for the immunoprecipitation of hypermethylated DNA [16,26]. Sequencing libraries of CVS and non-pregnant plasma samples were amplified for 30 cycles following MeDIP.

### Design and construction of target capture probes

Target capture probes (140-160bp) were designed to enrich selected fetal-specific DMRs across different chromosomes, as previously described [16]. Primers for each targeted region were designed to avoid repetitive and copy number variable regions. Capture probes were prepared using MyTaq HS DNA Polymerase (BioLine, London, UK) followed by purification as described previously [16,25]. Capture-probe concentrations were quantified using the NanoDrop spectrophotometer (Thermo Scientific, Wilmington, MA USA) and were pooled equimolarly prior for hybridization. Pooled probes were blunt-ended using the Quick Blunting kit (New England Biolabs) and biotinylated using the Quick Ligation Kit (NEB). Following purification (Qiagen) using the MinElute kit (Qiagen), probes were immobilized on streptavidin-coated magnetic Dynabeads M-270 (Thermo Scientific, Vilnius, Lithuania) [16,27].

### In-solution targeted enrichment

Target capture probes were used to enrich libraries using in-solution Hybridization [16,28]. Each barcoded library was mixed with 2x hybridization buffer (Agilent Technologies), 10x blocking agent (Agilent Technologies), blocking oligonucleotides, human Cot-1 (Invitrogen, Carlsbad, CA, USA) and salmon sperm DNA (Invitrogen) [16,25,28,29]. The libraries were then incubated with the biotinylated capture probes for 48 hours at 66°C and were eluted by heating [16,27]. Enriched fetal-specific regions were amplified for 12 cycles using Herculase II Fusion Enzyme kit (Agilent Technologies) and outward bound adaptor primers [27]. Following quantification with the KAPA Library Quantification Kit Illumina (KAPA Biosystems, Boston, MA, USA) the amplified post-captured libraries were sequenced on a HiSeq 2500 sequencing system platform (Illumina, San Diego, USA).

### Data analysis

#### NGS post-sequencing analysis

Adaptor sequences were trimmed with cutadapt v.1.2 and sequencing reads were aligned to the human reference genome GRCh37/hg19 (NCBI build 37) using the BWA v.0.7.4 MEM algorithm [30]. Following alignment the Picard tool was used to remove duplicate reads and convert aligned reads to a binary (BAM) file including only the uniquely aligned reads. Local realignment and base recalibration was performed with GATK and the SAMtools software was used to retrieve the read depth of each base [31,32].

#### Assessment of DMR variability

In order to assess the inter-individual methylation variability of selected DMRs in CVS and non-pregnant female plasma samples, we calculated their respective standard deviation and standard error for each DMR using their normalized read depth. Normalization was performed by equalizing the cumulative read depth of all DMRs across all samples. The 95% confidence intervals that were calculated provided information about the variability of each DMR. Additionally, by comparing their confidence intervals, the discrimination of CVS and non-pregnant female plasma samples methylation levels was derived for a given DMR. Potential overlap between the intervals suggests that there is no significant methylation difference between CVS and non-pregnant female plasma samples whereas lack of overlap suggests the possibility for discriminating the two tissues based on their methylation level.

#### Analysis of synthetic pregnancy samples and pregnancy cases

For the classification of pregnancies, a LOESS normalization model (non-parametric local polynomial regression model) was initially employed to alleviate GC-bias. The model was fitted only on regions that were not located on potentially trisomic chromosomes (i.e. chromosomes other than 13, 18 or 21) and the model’s predicted values were obtained for all regions. The normalized read depths were taken to be the ratio of the observed to the predicted read depths. Following the GC-normalization, the resulting normalized read depths were used in a two-sample t-test, where the DMRs of the potentially trisomic chromosome were compared to the remaining DMRs. The z-scores were calculated as a Welch's t-statistic test intended for use with two samples with potentially unequal variances.

## Results

### Inter-individual methylation variability

**Fig 1:**
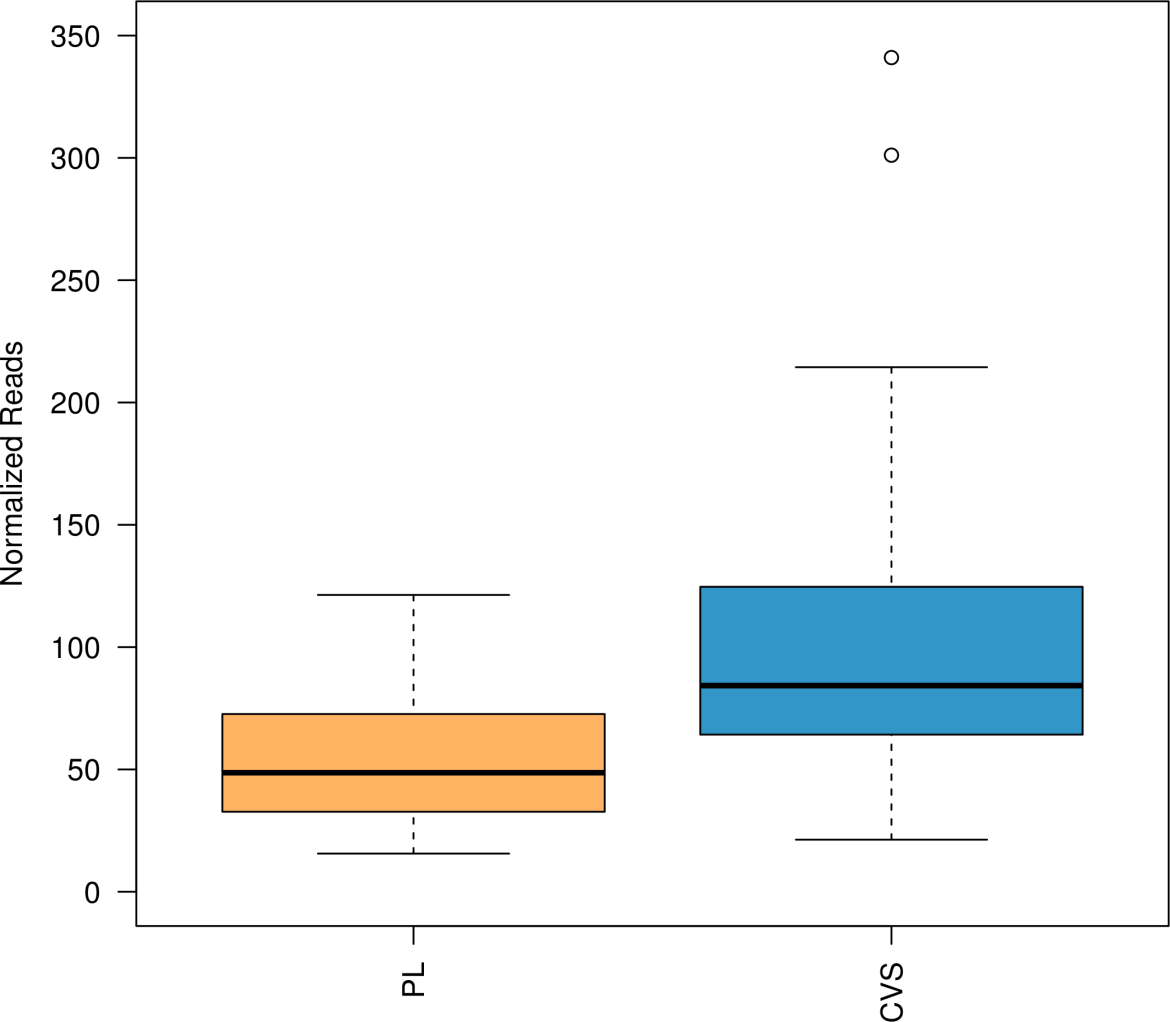
DMR methylation enrichment of the previously confirmed 331 fetal-specific DMRs in 29 CVS and 27 non-pregnant female plasma samples. Boxplots show the distribution of the enrichment (normalized reads) between the two tissues; plasma (orange) and CVS (blue). The boxplots were calculated from values across all samples and all DMRs. Methylation analysis showed significant difference in the enrichment between the two tissues (p<2e-16) with higher enrichment in CVS compared to female non-pregnant plasma samples. CVS: Chorionic Villus Samples, PL: female non-pregnant plasma sample.

Inter-individual methylation variability of the previously confirmed 331 fetal-specific DMRs [16] was ascertained in a cohort of 29 CVS and 27 non-pregnant female plasma samples using MeDIP in combination with in-solution targeted enrichment followed by NGS. Overall, methylation analysis for the 331 DMRs showed significantly higher enrichment in CVS as compared to non-pregnant female plasma samples (p<2e-16), confirming the hypermethylation of the selected DMRs in fetal tissue (Fig 1).

**Fig 2:**
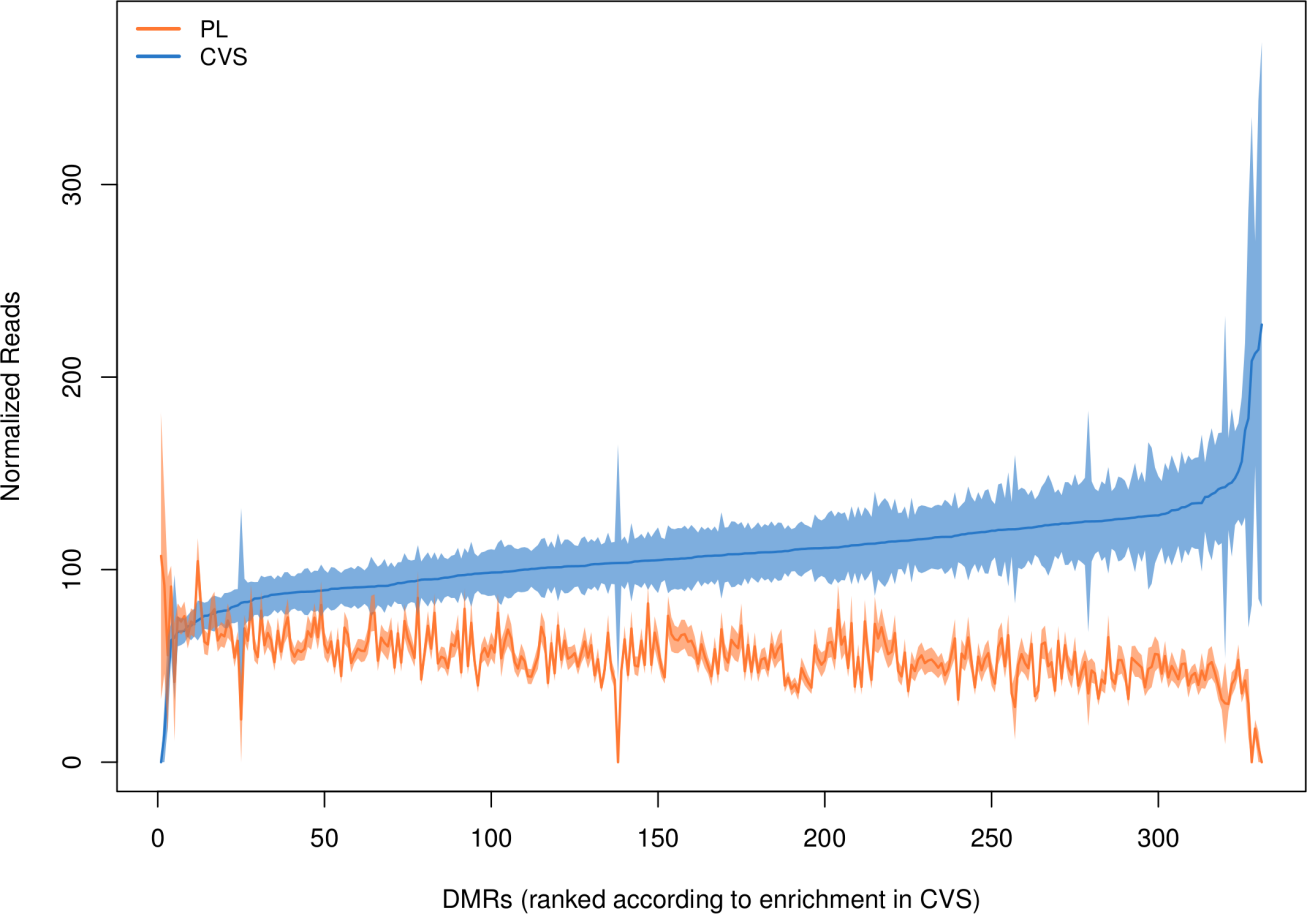
Methylation enrichment profile for all DMRs on CVS and female non-pregnant plasma samples. The DMRs are presented in the x-axis in order of increasing methylation in CVS. The blue line shows the mean enrichment in CVS (n = 29) for each DMR and the orange line the mean enrichment of each DMR in the plasma (n = 27). The shaded area around the mean shows the distance of plus and minus one standard error from the mean. Analysis indicated that the difference is statistically significant (p<0.001) in 313 out of 331 DMRs.

Pairwise methylation comparisons for each DMR between CVS and female plasma samples indicated that out of the 331 DMRs, 313 showed significant hypermethylation in CVS compared to plasma samples (p-value<0.001) (Fig 2).

The methylation enrichment in CVS was on average two fold higher compared to female plasma samples with mean enrichment values (normalized read depth) of 108 and 56 respectively (S1 Table). The coefficient of variation is a scale-independent metric and was chosen to assess the methylation variability of the CVS and non-pregnant female plasma samples for a given DMR. The coefficient of variation values of each DMR ranged from 0.44 to 5.39 for the CVS and from 0.32 to 5.2 for the female plasma samples. Methylation levels in CVS tissue appear to be more variable than in plasma samples with mean coefficient of variation values of 0.85 and 0.69 respectively (S1 Table). Despite the inter-individual variability, validated DMRs exhibited distinct and consistent methylation pattern in CVS and non-pregnant female plasma samples.

In order to identify DMRs that enable the best discrimination between fetal and maternal DNA, we first proceeded by adjusting the test’s p-values using the Tukey’s HSD method. A threshold of 0.05 was chosen for the adjusted p-values for differences between CVS and non-pregnant female plasma samples. In total 78 DMRs successfully passed the specific threshold (S1 Fig). For this subset of DMRs, the methylation enrichment in CVS was on average 2.6 fold higher compared to non-pregnant female plasma samples with mean enrichment values of 114 and 44 respectively. The coefficient of variation values of those DMRs ranged from 0.49 to 1.10 for the CVS and from 0.32 to 0.96 for the non-pregnant female plasma samples with mean coefficient of variation values of 0.74 and 0.58 respectively (S2 Table).

### Classification of trisomy 21 in synthetic pregnancy samples

**Fig 3:**
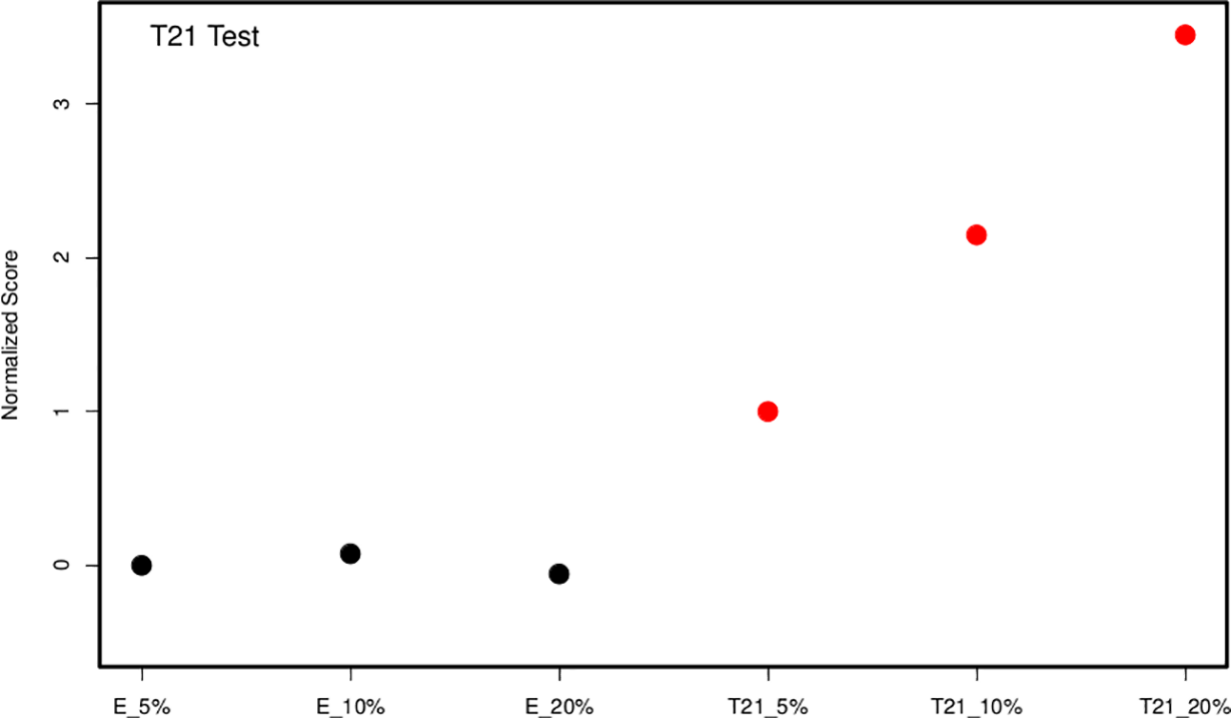
Detection of trisomy 21 in spiked-in samples. Euploid (black dots) and trisomy 21 (red dots) spiked-in samples simulating 5%, 10% and 20% fetal fractions were subjected to MeDIP combined with in-solution targeted enrichment followed by NGS. Z-score values of all trisomy 21 spiked-in samples were higher than the respective z-score values of euploid spiked-in samples and also demonstrated an increasing trend with respect to the fetal fraction. E, euploid; T21, Trisomy 21.

Towards the development of a NIPT assay for the detection of fetal aneuploidies, our initial efforts were focused on the implementation of MeDIP combined with in-solution targeted enrichment followed by NGS using three euploid and three trisomy 21 spiked-in samples simulating 5%, 10% and 20% fetal fractions. For the classification test, the read depth of each DMR was first normalized using a LOESS model on read depth against GC content in order to remove potential GC-bias in sequencing data. A z-test was then applied to each sample that compared the normalized read depth of DMRs on chr21 (region of interest) against the normalized read depth of DMRs on the other autosomes (reference). The median z-score of the three euploid spiked-in samples was subtracted from all six z-scores in order to center the euploid samples around the value of zero. Since a scale normalization was not appropriate with six samples, we did not use a hard threshold for classification, instead, this study assessed our ability to distinguish aneuploid samples in a series of dilutions. The z-scores of the six spiked-in samples showed a clear discrimination between the scores of the euploid and trisomy 21 samples (Fig 3).

In addition, there was a clear trend in the scores’ increase with respect to the fetal fraction of the aneuploid samples. Euploid samples remained unaffected by the increased fetal fraction.

### Classification of fetal aneuploidies in maternal plasma

Following the assessment of the NIPT assay on spiked-in samples, we tested its performance in a proof-of-concept study for the detection of fetal aneuploidies (trisomy 13, 18 and 21) in real pregnancy plasma samples. The study included 38 euploid and six aneuploid plasma samples (one trisomy 13, one trisomy 18 and four trisomy 21) obtained from women with singleton pregnancies (11–14 weeks of gestation). The observed fetal DNA fraction ranged from 5% to 16% with a median value of 11% (S3 Table).

**Fig 4:**
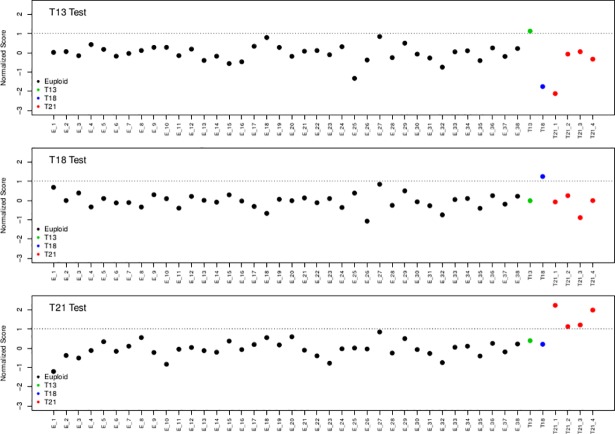
Detection of trisomies 13, 18 and 21 using pregnancy samples. Forty-four pregnancy samples including one trisomy 13 (green), one trisomy 18 (blue) and four trisomy 21 (red) samples were subjected to MeDIP combined with in-solution targeted enrichment followed by NGS. Trisomy 13 (a), trisomy 18 (b) and trisomy 21 (c) cases were successfully detected. All euploid cases (black dots) showed a normal representation of reads for the assessed chromosomes. E, euploid; T13, Trisomy 13; T18, Trisomy 18; T21, Trisomy 21.

GC-bias correction with LOESS and z-score calculation was performed as described in the Data Analysis section. Further score normalization was carried out by subtracting the median score value from the euploid samples, as described in the synthetic pregnancy samples. In addition, scale normalization was performed by dividing all z-scores by twice the standard deviation of the scores from the euploid samples. Therefore, a discriminating threshold of two standard deviations away from the median translated to a threshold value of 1. All four trisomy 21 as well as the trisomy 18 and trisomy 13 cases were correctly classified (normalized scores > 1). No false positive samples were detected (Fig 4).

## Discussion

An optimal panel of fetal-specific DMRs for NIPT should eliminate maternal background and show high homogeneity between individual fetuses [33]. Based on recent studies, the DNA methylation profile of specific DMRs exhibits stochastic variation among individuals [34,35]. In our previous study, 331 DMRs were selected and screened in a cohort of eight CVS and eight non-pregnant female plasma samples confirming the hypermethylation status of the selected DMRs. As a continuation of our previous work, in the current study, we investigated the methylation variability of the 331 previously confirmed DMRs in 29 CVS and 27 non-pregnant female plasma samples by combining MeDIP with in-solution targeted enrichment followed by NGS. All tested DMRs showed methylation variability among the different samples for both tissues. This is in agreement with several studies which attributed the methylation variability to a variety of factors including environmental conditions, as well as inter-experimental variability due to PCR-amplification bias and the presence of technical variability in MeDIP based assays [21,36–38]. It has also been suggested that non CpG-rich regions, characteristic of the tested DMRs, exhibit higher individual variability compared to CpG-rich regions [21,39]. Results also showed that CVS methylation exhibited higher variability than the plasma methylation (mean coefficient of variation 0.85 and 0.69 respectively). Remodeling of DNA methylation during different stages of embryonic development could explain the increased variability as the CVS samples used in this study were obtained at different weeks of gestation [40]. In addition, cell heterogeneity of CVS tissue, consisting of a mixture of syncytiotrophoblastic, cytotrophoblastic, mesodermal and fetal endothelial/vascular cells, can contribute to the high tissue variability as the methylation value obtained is a reflection of the methylation status of all the different cell populations [41].

Overall methylation analysis showed that despite the inter-individual methylation variability, the tested DMRs showed differential enrichment between fetal DNA (CVS) and maternal DNA (non-pregnant female plasma sample). Specifically, CVS samples showed statistically significant hypermethylation (p<2e-16) as compared to plasma samples with mean enrichment values of 108 and 56 respectively. Additional statistical analysis of the methylation status of the initial set of 331 DMRs revealed that 78 DMRs (adjusted p-value<0.05) showed the best discrimination between fetal and maternal tissues. Based on our statistical classification model this number is not sufficient for clinical NIPT applications, especially considering the limited number of DMRs in the potentially aneuploid regions (ten DMRs on chromosome 21, four DMRs on chromosome 18 and three DMRs on chromosome 13). Even though a small subset of potential DMRs exhibited p-values higher than our specified threshold (p = 0.001) we still included them in our assay since even for these markers the extra copy of a potential trisomic sample can still contribute towards the classification power of the test.

Additionally, we developed and assessed the performance of a novel NIPT assay for the detection of the most common fetal aneuploidies during pregnancy (trisomy 13, 18 and 21). For the first time, we implemented MeDIP in combination with in-solution hybridization on spiked-in and pregnancy plasma samples which resulted to the successful classification of all euploid and aneuploid samples. Specifically, implementation of our double enrichment approach on spiked-in samples detected all trisomy 21 spiked-in samples as they showed higher z-scores values than the respective z-score values of euploid spiked-in samples. Subsequently, the performance of this novel method was evaluated on pregnancy cases for aneuploidy detection. This proof-of-concept study provided an accurate detection of all trisomy 21 (n = 4), trisomy 18 (n = 1) and trisomy 13 (n = 1) samples using a total of 44 pregnancy samples whereas no false positive samples were observed. This novel assay provides the starting point for the development of an alternative and cost-effective non-invasive method for the detection of fetal aneuploidy from maternal plasma. Our approach increases the diagnostic accuracy as it enables a very high read depth and robust analysis of the targeted DMRs. In addition, the targeted enrichment of multiple DMRs reduces the sequencing cost per sample as it enables a higher number of samples to be analyzed simultaneously.

The classification of fetal trisomies was performed using 98 markers (39, 31 and 28 DMRs on chromosomes 13, 18 and 21 respectively) (S1 Table). Future work should focus on the discovery of additional DMRs on these chromosomes in order to increase the statistical classification power of our model. All tested DMRs are located throughout the genome therefore, using refined criteria, we can further increase their number by identifying markers on critical regions of microdeletion syndromes and clinically relevant point mutations. This will expand the disease panel of our assay and offer more choices for couples to make informed decision regarding their pregnancy.

Overall, the method described in this study provides unbiased and accurate results for a wide range of cfDNA concentrations at different fetal fractions, ranging from 5–16%. In our proof-of-concept study we have correctly classified all euploid and aneuploid cases. Previous studies have utilized a large number of samples for validation purposes [42,43]. Thus, as the scope of our assay is its clinical implementation, it is essential that a large validation study is performed, using additional affected pregnancy plasma samples in order to assess the diagnostic sensitivity and specificity of the assay.

## Conclusion

Investigation of inter-individual methylation variability on selected DMRs showed that despite the presence of variability, there is a distinct and robust difference between fetal and maternal tissues. In addition, we are reporting for the first time the development of a novel assay for the detection of fetal aneuploidies from maternal plasma samples, using MeDIP in combination with in-solution targeted enrichment followed by NGS. In a proof-of-concept study presented herein, our assay correctly classified all euploid and aneuploid cases without any false positive results (n = 44). However, a larger validation study including more aneuploid samples and more DMRs should be performed to further assess the diagnostic sensitivity and specificity of the assay. In addition, the assay’s targeted nature significantly reduces the analysis cost per sample while providing high read depth at regions of interest increasing significantly its accuracy.

Conclusively, our work provides the starting point for the development of a clinical NIPT assay for the detection of fetal aneuploidies using a robust and well-characterized set of fetal specific biomarkers and provides the possibility for the expansion of the existing disease panel to include detection of microdeletion syndromes and potentially monogenic diseases.

## Supporting information

S1 Fig Inter-individual methylation variability of CVS and non-pregnant female plasma samples for the 78 DMRs. Squares show the mean methylation enrichment for the CVS (blue) and non-pregnant female plasma samples (orange). The extended lines show the distance of one standard error (plus and minus). All DMRs exhibited distinct and consistently higher methylation levels among the 29 CVS samples as compared to the 27 non-pregnant female plasma samples. The 78 markers were chosen so their Tukey HSD adjusted p-values are less than 0.05.
(TIFF)

S1 Table Enrichment values and coefficient of variation of 331 validated DMRs. (DOCX)

S2 Table Enrichment values of 78 validated DMRs obtained from MeDIP-combined with a targeted hybridization method followed by NGS. (DOCX)

S3 Table Fetal fraction estimation of pregnancy cases. (DOCX)

## References

[1] Akolekar R, Beta J, Picciarelli G, Ogilvie C, D’Antonio F. Procedure-related risk of miscarriage following amniocentesis and chorionic villus sampling: A systematic review and meta-analysis. Ultrasound Obstet Gynecol. 2015;45(1):16–26. doi: 10.1002/uog.14636 25042845 10.1002/uog.14636

[2] Lo YMD, Corbetta N, Chamberlain PF, Rai V, Sargent IL, Redman CWG. Early report Presence of fetal DNA in maternal plasma and serum. Lancet. 1997;350(9076):485–7. doi: 10.1016/S0140-6736(97)02174-0 9274585 10.1016/S0140-6736(97)02174-0

[3] Motavaf M, Sadeghizadeh M. Noninvasive Prenatal Test by Cell-Free Fetal DNA in Maternal Plasma: Current Progress and Prospective Clinical Applications. 2014;5(3):3–8.

[4] Ng EKO, Tsui NBY, Lau TK, Leung TN, Chiu RWK, Panesar NS, et al mRNA of placental origin is readily detectable in maternal plasma. Proc Natl Acad Sci U S A. 2003;100(8):4748–53. doi: 10.1073/pnas.0637450100 12644709 10.1073/pnas.0637450100 PMC153627

[5] Avent ND, Plummer ZE, Madgett TE, Maddocks DG, Soothill PW. Post-genomics studies and their application to non-invasive prenatal diagnosis. Semin Fetal Neonatal Med. 2008;13(2):91–8. doi: 10.1016/j.siny.2007.12.011 18249591 10.1016/j.siny.2007.12.011

[6] Lo YM, Tein MS, Lau TK, Haines CJ, Leung TN, Poon PM, et al Quantitative analysis of fetal DNA in maternal plasma and serum: implications for noninvasive prenatal diagnosis. Am J Hum Genet. 1998;62(4):768–75. doi: 10.1086/301800 9529358 10.1086/301800 PMC1377040

[7] Lo YMD, Tsui NBY, Chiu RWK, Lau TK, Leung TN, Heung MMS, et al Plasma placental RNA allelic ratio permits noninvasive prenatal chromosomal aneuploidy detection. Nat Med. 2007;13(2):218–23. doi: 10.1038/nm1530 17206148 10.1038/nm1530

[8] Chiu RWK, Chan KCA, Gao Y, Lau VYM, Zheng W, Leung TY, et al Noninvasive prenatal diagnosis of fetal chromosomal aneuploidy by massively parallel genomic sequencing of DNA in maternal plasma. Proc Natl Acad Sci U S A. 2008 23;105(51):20458–63. doi: 10.1073/pnas.0810641105 19073917 10.1073/pnas.0810641105 PMC2600580

[9] Fan HC, Blumenfeld YJ, Chitkara U, Hudgins L, Quake SR. Noninvasive diagnosis of fetal aneuploidy by shotgun sequencing DNA from maternal blood. Proc Natl Acad Sci U S A. 2008;105(42):16266–71. doi: 10.1073/pnas.0808319105 18838674 10.1073/pnas.0808319105 PMC2562413

[10] Sparks AB, Struble CA, Wang ET, Song K, Oliphant A. Noninvasive prenatal detection and selective analysis of cell-free DNA obtained from maternal blood: Evaluation for trisomy 21 and trisomy 18. Am J Obstet Gynecol. 2012;206(4):319.e1–319.e9. 22464072 10.1016/j.ajog.2012.01.030

[11] Sparks AB, Wang ET, Struble CA, Barrett W, Stokowski R, Mcbride C, et al Selective analysis of cell-free DNA in maternal blood for evaluation of fetal trisomy. Prenat Diagn. 2012;32(1):3–9. doi: 10.1002/pd.2922 22223233 10.1002/pd.2922 PMC3500507

[12] Liao GJW, Lun FMF, Zheng YWL, Chan KCA, Leung TY, Lau TK, et al Targeted massively parallel sequencing of maternal plasma DNA permits efficient and unbiased detection of fetal alleles. Clin Chem. 2011;57(1):92–101. doi: 10.1373/clinchem.2010.154336 21078840 10.1373/clinchem.2010.154336

[13] Papageorgiou E a, Karagrigoriou A, Tsaliki E, Velissariou V, Carter NP, Patsalis PC. Fetal-specific DNA methylation ratio permits noninvasive prenatal diagnosis of trisomy 21. Nat Med. 2011;17(4):510–3. doi: 10.1038/nm.2312 21378977 10.1038/nm.2312 PMC3977039

[14] Tsaliki E, Papageorgiou E a., Spyrou C, Koumbaris G, Kypri E, Kyriakou S, et al MeDIP real-time qPCR of maternal peripheral blood reliably identifies trisomy 21. Prenat Diagn. 2012;32(10):996–1001. doi: 10.1002/pd.3947 22833530 10.1002/pd.3947

[15] Lee DE, Kim SY, Lim JH, Park SY, Ryu HM. Non-invasive prenatal testing of trisomy 18 by an epigenetic marker in first trimester maternal plasma. PLoS One. 2013;8(11). 10.1371/journal.pone.0078136 PMC3815335 24223769

[16] Keravnou A, Ioannides M, Tsangaras K, Loizides C, Hadjidaniel MD, Papageorgiou EA, et al Whole genome fetal and maternal DNA methylation analysis using MeDIP-NGS for the identification of differentially methylated regions. Genet Res (Camb). 2016;98:1–9. 10.1017/S0016672316000136 PMC6865150 27834155

[17] Chim SSC, Jin S, Lee TYH, Lun FMF, Lee WS, Chan LYS, et al Systematic search for placental DNA-methylation markers on chromosome 21: Toward a maternal plasma-based epigenetic test for fetal trisomy 21. Clin Chem. 2008;54(3):500–11. doi: 10.1373/clinchem.2007.098731 18202156 10.1373/clinchem.2007.098731

[18] Old RW, Crea F, Puszyk W, Hultén MA. Candidate epigenetic biomarkers for non-invasive prenatal diagnosis of Down syndrome. Reprod Biomed Online. 2007;15(2):227–35. 17697502 10.1016/s1472-6483(10)60713-4

[19] Chu T, Burke B, Bunce K, Surti U, Allen Hogge W, Peters DG. A microarray-based approach for the identification of epigenetic biomarkers for the noninvasive diagnosis of fetal disease. Prenat Diagn. 2009;29(11):1020–30. doi: 10.1002/pd.2335 19650061 10.1002/pd.2335

[20] Papageorgiou E a, Fiegler H, Rakyan V, Beck S, Hulten M, Lamnissou K, et al Sites of differential DNA methylation between placenta and peripheral blood: molecular markers for noninvasive prenatal diagnosis of aneuploidies. Am J Pathol. 2009;174(5):1609–18. doi: 10.2353/ajpath.2009.081038 19349366 10.2353/ajpath.2009.081038 PMC2671250

[21] Ioannides M, Papageorgiou E a, Keravnou A, Tsaliki E, Spyrou C, Hadjidaniel M, et al Inter-individual methylation variability in differentially methylated regions between maternal whole blood and first trimester CVS. Mol Cytogenet. 2014;7(73):2–8. 25426166 10.1186/s13039-014-0073-8 PMC4243368

[22] Huang DJ, Mergenthaler-Gatfield S, Hahn S, Holzgreve W, Zhong XY. Isolation of cell-free DNA from maternal plasma using manual and automated systems. Methods Mol Biol. 2008;444(2):203–8. 18425482 10.1007/978-1-59745-066-9_15

[23] Zimmermann B, El-Sheikhah A, Nicolaides K, Holzgreve W, Hahn S. Optimized real-time quantitative PCR measurement of male fetal DNA in maternal plasma. Clin Chem. 2005;51(9):1598–604. doi: 10.1373/clinchem.2005.051235 16020496 10.1373/clinchem.2005.051235

[24] Meyer M, Kircher M. Illumina sequencing library preparation for highly multiplexed target capture and sequencing. Cold Spring Harb Protoc. 2010;5(6):1–10. 10.1101/pdb.prot5448 20516186

[25] Koumbaris G, Kypri E, Tsangaras K, Achilleos A, Mina P., Neofytou M. & Patsalis PC. Cell-free DNA analysis of targeted genomic regions in maternal plasma for non- invasive prenatal testing of trisomy 21, trisomy 18, trisomy 13 and fetal gender. Clin Chem. 2016;62(6):848–855. doi: 10.1373/clinchem.2015.252502 27117469 10.1373/clinchem.2015.252502

[26] Borgel J, Guibert S, Weber M. Methylated DNA Immunoprecipitation (MeDIP) from Low Amounts of Cells. Engel N, editor. Methods Mol Biol. 2012;925:149–58. doi: 10.1007/978-1-62703-011-3_9 22907495 10.1007/978-1-62703-011-3_9

[27] Tsangaras K, Siracusa MC, Nikolaidis N, Ishida Y, Cui P, Vielgrader H, et al Hybridization capture reveals evolution and conservation across the entire koala retrovirus genome. PLoS One. 2014;9(4):1–14. 10.1371/journal.pone.0095633 PMC3994108 24752422

[28] Maricic T, Whitten M, Pääbo S. Multiplexed DNA sequence capture of mitochondrial genomes using PCR products. PLoS One. 2010;5(11):1–5. 10.1371/journal.pone.0014004 PMC2982832 21103372

[29] Neofytou MC, Tsangaras K, Kypri E, Loizides C, Ioannides M, Achilleos A, et al Targeted capture enrichment assay for non-invasive prenatal testing of large and small size sub-chromosomal deletions and duplications. PLoS One. 2017;12(2):1–13. 10.1371/journal.pone.0171319 PMC5291539 28158220

[30] Li H, Durbin R. Fast and accurate short read alignment with Burrows-Wheeler transform. Bioinformatics. 2009;25(14):1754–60. doi: 10.1093/bioinformatics/btp324 19451168 10.1093/bioinformatics/btp324 PMC2705234

[31] McKenna A, Hanna M, Banks E, Sivachenko A, Cibulskis K, Kernytsky A, et al The Genome Analysis Toolkit: A MapReduce framework for analyzing next-generation DNA sequencing data. Genome Res. 2010;20(9):1297–303. doi: 10.1101/gr.107524.110 20644199 10.1101/gr.107524.110 PMC2928508

[32] Li H, Handsaker B, Wysoker A, Fennell T, Ruan J, Homer N, et al The Sequence Alignment/Map format and SAMtools. Bioinformatics. 2009;25(16):2078–9. doi: 10.1093/bioinformatics/btp352 19505943 10.1093/bioinformatics/btp352 PMC2723002

[33] Xiang Y, Zhang J, Li Q, Zhou X, Wang T, Xu M, et al DNA methylome profiling of maternal peripheral blood and placentas reveal potential fetal DNA markers for non-invasive prenatal testing. Mol Hum Reprod. 2014;20(9):875–84. doi: 10.1093/molehr/gau048 24996894 10.1093/molehr/gau048

[34] Schneider E, Pliushch G, El Hajj N, Galetzka D, Puhl A, Schorsch M, et al Spatial, temporal and interindividual epigenetic variation of functionally important DNA methylation patterns. Nucleic Acids Res. 2010;38(12):3880–90. doi: 10.1093/nar/gkq126 20194112 10.1093/nar/gkq126 PMC2896520

[35] Xie H, Wang M, de Andrade A, Bonaldo MDF, Galat V, Arndt K, et al Genome-wide quantitative assessment of variation in DNA methylation patterns. Nucleic Acids Res. 2011;39(10):4099–108. doi: 10.1093/nar/gkr017 21278160 10.1093/nar/gkr017 PMC3105398

[36] Butcher LM, Beck S. AutoMeDIP-seq: a high-throughput, whole genome, DNA methylation assay. Methods. 2010;52(3):223–31. doi: 10.1016/j.ymeth.2010.04.003 20385236 10.1016/j.ymeth.2010.04.003 PMC2977854

[37] Wong CCY, Caspi A, Williams B, Craig IW, Houts R, Ambler A, et al A longitudinal study of epigenetic variation in twins. Epigenetics. 2010;5(6):516–26. doi: 10.4161/epi.5.6.12226 20505345 10.4161/epi.5.6.12226 PMC3322496

[38] Lam LL, Emberly E, Fraser HB, Neumann SM, Chen E, Miller GE, et al Factors underlying variable DNA methylation in a human community cohort. Proc Natl Acad Sci. 2012;109(Supplement_2):17253–60. 23045638 10.1073/pnas.1121249109 PMC3477380

[39] Bock C, Walter J, Paulsen M, Lengauer T. Inter-individual variation of DNA methylation and its implications for large-scale epigenome mapping. Nucleic Acids Res. 2008;36(10):2–11. 10.1093/nar/gkn122 PMC2425484 18413340

[40] Slieker RC, Roost MS, van Iperen L, Suchiman HED, Tobi EW, Carlotti F, et al DNA Methylation Landscapes of Human Fetal Development. PLoS Genet. 2015;11(10):1–19. 10.1371/journal.pgen.1005583 PMC4619663 26492326

[41] Hatt L, Aagaard MM, Graakjaer J, Bach C, Sommer S, Agerholm IE, et al Microarray-Based Analysis of Methylation Status of CpGs in Placental DNA and Maternal Blood DNA–Potential New Epigenetic Biomarkers for Cell Free Fetal DNA-Based Diagnosis. Chan KYK, editor. PLoS One. 2015;10(7):1–12. 10.1371/journal.pone.0128918 PMC4521692 26230497

[42] Lefkowitz RB, Tynan JA, Liu T, Wu Y, Mazloom AR, Almasri E, et al Clinical validation of a noninvasive prenatal test for genomewide detection of fetal copy number variants. Am J Obstet Gynecol. 2016;215(2):227.e1-227.e16. 26899906 10.1016/j.ajog.2016.02.030

[43] Gerundino F, Giachini C, Contini E, Benelli M, Marseglia G, Giuliani C, et al Validation of a method for noninvasive prenatal testing for fetal aneuploidies risk and considerations for its introduction in the Public Health System. J Matern Fetal Neonatal Med. 2016;30(6):1–7. 27226231 10.1080/14767058.2016.1183633

