# Supplementary figures and images for: MeDIP combined with in-solution targeted enrichment followed by NGS: Inter-individual methylation variability of fetal-specific biomarkers and their implementation in a proof of concept study for NIPT
Source: PLoS One. 2018 Jun 11;13(6):e0199010. doi: 10.1371/journal.pone.0199010 (PMC5995407; doi:10.1371/journal.pone.0199010)

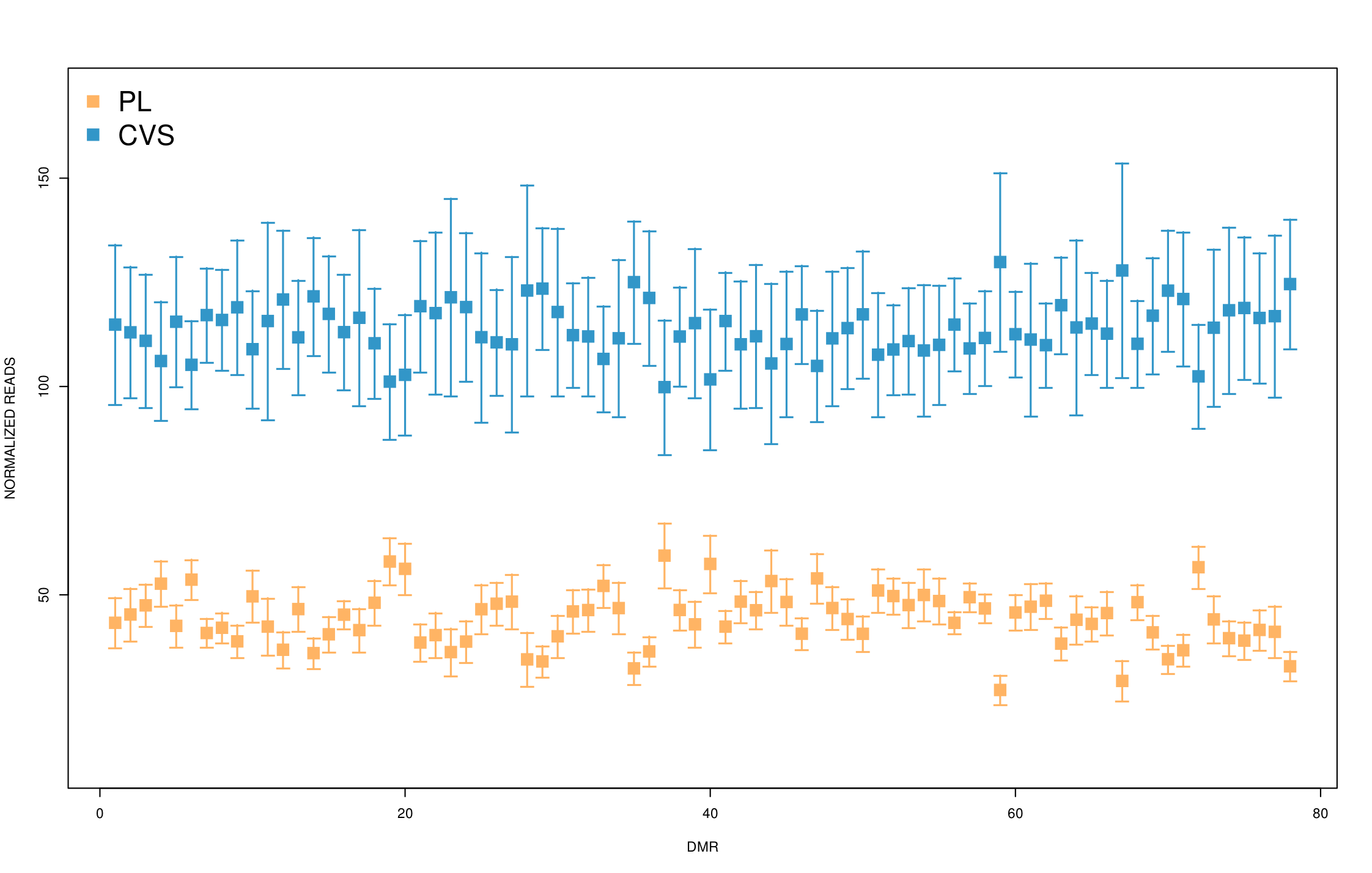

Supplement: S1 Fig — Squares show the mean methylation enrichment for the CVS (blue) and non-pregnant female plasma samples (orange). The extended lines show the distance of one standard error (plus and minus). All DMRs exhibited distinct and consistently higher methylation levels among the 29 CVS samples as compared to the 27 non-pregnant female plasma samples. The 78 markers were chosen so their Tukey HSD adjusted p-values are less than 0.05. (TIFF) [file pone.0199010.s001.tiff]
