# Supplementary material for: MeDIP combined with in-solution targeted enrichment followed by NGS: Inter-individual methylation variability of fetal-specific biomarkers and their implementation in a proof of concept study for NIPT
Source: PLoS One. 2018 Jun 11;13(6):e0199010. doi: 10.1371/journal.pone.0199010 (PMC5995407; doi:10.1371/journal.pone.0199010)
Supplement: S1 Table — (DOCX) [file pone.0199010.s002.docx]

| **DMR** | **Chromosome** | **Location** | **Mean CVS** | **Mean PL** | **SD CVS** | **SD PL** | **CV CVS** | **CV PL** |
| --- | --- | --- | --- | --- | --- | --- | --- | --- |
| 1 | chr1 | 1p36.31 | 80.4 | 69 | 44.2 | 34.6 | 0.55 | 0.5 |
| 2 | chr1 | 1p36.23 | 124.7 | 47 | 112.1 | 34.1 | 0.9 | 0.72 |
| 3 | chr1 | 1p36.22 | 121.8 | 48.8 | 91.4 | 35.3 | 0.75 | 0.72 |
| 4 | chr1 | 1p36.13 | 126.3 | 53.1 | 118.5 | 41.3 | 0.94 | 0.78 |
| 5 | chr1 | 1p36.13 | 111.3 | 61.9 | 97.8 | 40.8 | 0.88 | 0.66 |
| 6 | chr1 | 1p36.13 | 108.9 | 46.6 | 84.6 | 25.8 | 0.78 | 0.55 |
| 7 | chr1 | 1p36.11 | 109 | 54.1 | 79.1 | 29.2 | 0.73 | 0.54 |
| 8 | chr1 | 1p34.3 | 116.1 | 52.8 | 88 | 31.5 | 0.76 | 0.6 |
| 9 | chr1 | 1p34.3 | 142.9 | 30.6 | 478.4 | 110.4 | 3.35 | 3.61 |
| 10 | chr1 | 1p34.2 | 125.1 | 46.1 | 91.2 | 28.4 | 0.73 | 0.62 |
| 11 | chr1 | 1p34.1 | 64.7 | 41.7 | 175.6 | 158.9 | 2.72 | 3.81 |
| 12 | chr1 | 1p34.1 | 145.2 | 40.5 | 207.3 | 32 | 1.43 | 0.79 |
| 13 | chr1 | 1p33 | 105.4 | 76 | 94.9 | 52.7 | 0.9 | 0.69 |
| 14 | chr1 | 1p32.3 | 113.5 | 71.9 | 145.7 | 73.5 | 1.28 | 1.02 |
| 15 | chr1 | 1p32.3 | 123.3 | 51.9 | 118.1 | 41.6 | 0.96 | 0.8 |
| 16 | chr1 | 1p32.1 | 105.6 | 63.3 | 86.2 | 34.9 | 0.82 | 0.55 |
| 17 | chr1 | 1p22.2 | 208.3 | NA | 682.3 | 0 | 3.28 | NA |
| 18 | chr1 | 1p22.2 | 91.5 | 61.9 | 75.3 | 34 | 0.82 | 0.55 |
| 19 | chr1 | 1p22.2 | 120.6 | 59.7 | 127.2 | 46.6 | 1.05 | 0.78 |
| 20 | chr1 | 1p21.3 | 95.9 | 48.9 | 51.6 | 22.8 | 0.54 | 0.47 |
| 21 | chr1 | 1p21.2 | 111 | 38.7 | 57.7 | 16.9 | 0.52 | 0.44 |
| 22 | chr1 | 1p12 | 129 | 55.1 | 142.2 | 40.7 | 1.1 | 0.74 |
| 23 | chr1 | 1q21.2 | 118.3 | 56.4 | 83 | 38.3 | 0.7 | 0.68 |
| 24 | chr1 | 1q22 | 227.3 | NA | 789.4 | 0 | 3.47 | NA |
| 25 | chr1 | 1q23.1 | 116.3 | 53.3 | 84.6 | 32.7 | 0.73 | 0.61 |
| 26 | chr1 | 1q24.1 | 178.6 | 30.6 | 584.8 | 93 | 3.28 | 3.04 |
| 27 | chr1 | 1q24.2 | 109.2 | 54.2 | 72.3 | 29 | 0.66 | 0.53 |
| 28 | chr1 | 1q25.3 | 105.5 | 63.8 | 83.6 | 37.2 | 0.79 | 0.58 |
| 29 | chr1 | 1q31.3 | 109.7 | 39.8 | 61.6 | 17.9 | 0.56 | 0.45 |
| 30 | chr1 | 1q41 | 119.1 | 38.9 | 87.1 | 20.5 | 0.73 | 0.53 |
| 31 | chr1 | 1q41 | 127.9 | 48.4 | 206 | 57.3 | 1.61 | 1.18 |
| 32 | chr1 | 1q41 | 111.1 | 50.6 | 77.4 | 33.1 | 0.7 | 0.65 |
| 33 | chr1 | 1q42.3 | 102.9 | 53.4 | 75.7 | 30.1 | 0.74 | 0.56 |
| 34 | chr1 | 1q44 | 81.4 | 63 | 45.7 | 26.2 | 0.56 | 0.41 |
| 35 | chr2 | 2p21 | 137.9 | 50.5 | 151.9 | 42.4 | 1.1 | 0.84 |
| 36 | chr2 | 2p14 | 70.6 | 65.6 | 30.9 | 34 | 0.44 | 0.52 |
| 37 | chr2 | 2p14 | 104.3 | 49.7 | 67.7 | 26.6 | 0.65 | 0.54 |
| 38 | chr2 | 2p14 | 125.6 | 65 | 149.1 | 59.1 | 1.19 | 0.91 |
| 39 | chr2 | 2p13.3 | 127.7 | 38.9 | 94.3 | 23.8 | 0.74 | 0.61 |
| 40 | chr2 | 2q24.2 | 101.7 | 53.8 | 74.9 | 31.2 | 0.74 | 0.58 |
| 41 | chr2 | 2q31.1 | 91.2 | 77.7 | 68.4 | 49.3 | 0.75 | 0.63 |
| 42 | chr2 | 2q32.3 | 107.2 | 44.6 | 70.9 | 26.7 | 0.66 | 0.6 |
| 43 | chr2 | 2q36.1 | 95.1 | 77.6 | 75 | 51.5 | 0.79 | 0.66 |
| 44 | chr2 | 2q37.1 | 120.9 | 35.8 | 76 | 18.8 | 0.63 | 0.52 |
| 45 | chr3 | 3p25.1 | 124.5 | 39.7 | 104.1 | 27.2 | 0.84 | 0.68 |
| 46 | chr3 | 3p22.1 | 91.5 | 64.7 | 60.6 | 35.4 | 0.66 | 0.55 |
| 47 | chr3 | 3p14.3 | 125.8 | 43.4 | 80.5 | 23.5 | 0.64 | 0.54 |
| 48 | chr3 | 3p14.3 | 99.9 | 55 | 62.5 | 34.5 | 0.63 | 0.63 |
| 49 | chr3 | 3p14.2 | 105.8 | 66.5 | 91.8 | 38.7 | 0.87 | 0.58 |
| 50 | chr3 | 3p14.1 | 103.8 | 69.4 | 109.5 | 46.6 | 1.05 | 0.67 |
| 51 | chr3 | 3q26.32 | 109.7 | 43.9 | 72.3 | 16.7 | 0.66 | 0.38 |
| 52 | chr3 | 3q27.1 | 130.9 | 46.7 | 127.8 | 30.3 | 0.98 | 0.65 |
| 53 | chr3 | 3q28 | 128 | 49.5 | 189.9 | 54.8 | 1.48 | 1.11 |
| 54 | chr3 | 3q29 | 118.8 | 64.8 | 131.3 | 55.9 | 1.1 | 0.86 |
| 55 | chr4 | 4p15.33 | 115.8 | 55.5 | 94.8 | 34.9 | 0.82 | 0.63 |
| 56 | chr4 | 4p15.1 | 108 | 61 | 91.1 | 41.8 | 0.84 | 0.68 |
| 57 | chr4 | 4p14 | 108.4 | 47.3 | 69.9 | 27.5 | 0.64 | 0.58 |
| 58 | chr4 | 4p13 | 90 | 62.7 | 60.8 | 31.4 | 0.68 | 0.5 |
| 59 | chr4 | 4q21.23 | 123.1 | 49.8 | 141.3 | 33.6 | 1.15 | 0.67 |
| 60 | chr4 | 4q21.23 | 112.2 | 48.9 | 70.2 | 24.1 | 0.63 | 0.49 |
| 61 | chr4 | 4q26 | 95.5 | 54.8 | 70.3 | 27.7 | 0.74 | 0.51 |
| 62 | chr4 | 4q28.1 | 108.4 | 60.9 | 86.6 | 37.4 | 0.8 | 0.61 |
| 63 | chr4 | 4q31.1 | 107.3 | 58.7 | 81.5 | 33.3 | 0.76 | 0.57 |
| 64 | chr4 | 4q31.3 | 99 | 68.9 | 75.1 | 44.5 | 0.76 | 0.65 |
| 65 | chr4 | 4q32.1 | 109.1 | 61.2 | 86.1 | 36.1 | 0.79 | 0.59 |
| 66 | chr4 | 4q35.1 | 119.5 | 47.8 | 91.5 | 32 | 0.77 | 0.67 |
| 67 | chr5 | 5p13.1 | 126.1 | 40.8 | 89.6 | 24.4 | 0.71 | 0.6 |
| 68 | chr5 | 5q11.1 | 121.4 | 56.1 | 114.1 | 45.1 | 0.94 | 0.8 |
| 69 | chr5 | 5q14.1 | 106.8 | 51.1 | 67.2 | 25.7 | 0.63 | 0.5 |
| 70 | chr5 | 5q14.1 | 121.1 | 50.6 | 108.3 | 35.7 | 0.89 | 0.71 |
| 71 | chr5 | 5q15 | 125.1 | 47.7 | 112.1 | 32.8 | 0.9 | 0.69 |
| 72 | chr5 | 5q23.2 | 111.1 | 60.7 | 94.8 | 45 | 0.85 | 0.74 |
| 73 | chr5 | 5q31.1 | 103.5 | 47.3 | 63 | 22.5 | 0.61 | 0.48 |
| 74 | chr5 | 5q31.1 | 93.3 | 73.3 | 82.1 | 45.5 | 0.88 | 0.62 |
| 75 | chr5 | 5q31.3 | 88.5 | 57.2 | 52.4 | 24.2 | 0.59 | 0.42 |
| 76 | chr5 | 5q33.2 | 117.6 | 64.3 | 122.2 | 52.3 | 1.04 | 0.81 |
| 77 | chr5 | 5q34 | 117 | 56.2 | 85.1 | 36.5 | 0.73 | 0.65 |
| 78 | chr5 | 5q35.1 | 130.8 | 50 | 154.2 | 38.3 | 1.18 | 0.77 |
| 79 | chr6 | 6p24.3 | 94.4 | 85.3 | 99 | 71.8 | 1.05 | 0.84 |
| 80 | chr6 | 6p24.1 | 86.9 | 63.4 | 54 | 34 | 0.62 | 0.54 |
| 81 | chr6 | 6p22.3 | 125.2 | 42.9 | 111.4 | 29.9 | 0.89 | 0.7 |
| 82 | chr6 | 6p22.3 | 86 | 60.7 | 54.3 | 27.9 | 0.63 | 0.46 |
| 83 | chr6 | 6p12.3 | 99.3 | 52.1 | 63.7 | 25.9 | 0.64 | 0.5 |
| 84 | chr6 | 6p12.3 | 98.3 | 54.8 | 64.5 | 29.1 | 0.66 | 0.53 |
| 85 | chr6 | 6p12.3 | 93.9 | 60 | 58.1 | 31.2 | 0.62 | 0.52 |
| 86 | chr6 | 6q16.3 | 101.2 | 71 | 94.7 | 39.9 | 0.94 | 0.56 |
| 87 | chr6 | 6q21 | 109.4 | 61.4 | 80 | 41.3 | 0.73 | 0.67 |
| 88 | chr6 | 6q21 | 133.3 | 39.8 | 140.1 | 32.3 | 1.05 | 0.81 |
| 89 | chr6 | 6q21 | 125.5 | 40.9 | 101.3 | 27.2 | 0.81 | 0.67 |
| 90 | chr6 | 6q23.3 | 125 | 52 | 122.3 | 34.4 | 0.98 | 0.66 |
| 91 | chr6 | 6q25.3 | 115 | 56.9 | 95.8 | 36.8 | 0.83 | 0.65 |
| 92 | chr7 | 7p21.1 | 114.5 | 56.1 | 110.6 | 37.9 | 0.97 | 0.67 |
| 93 | chr7 | 7p21.1 | 103.5 | 63.8 | 73.7 | 36 | 0.71 | 0.56 |
| 94 | chr7 | 7p15.3 | 108.5 | 47 | 67.3 | 25.9 | 0.62 | 0.55 |
| 95 | chr7 | 7p15.1 | 89.1 | 81.6 | 73.1 | 63 | 0.82 | 0.77 |
| 96 | chr7 | 7p14.3 | 87.2 | 68.2 | 53.2 | 35.8 | 0.61 | 0.53 |
| 97 | chr7 | 7p14.3 | 119.3 | 54.8 | 112.9 | 37.5 | 0.95 | 0.68 |
| 98 | chr7 | 7p14.2 | 83.2 | 69.5 | 65.3 | 36.1 | 0.79 | 0.52 |
| 99 | chr7 | 7p14.2 | 120.9 | 65.9 | 159.4 | 55.8 | 1.32 | 0.85 |
| 100 | chr7 | 7p14.2 | 112.7 | 54.7 | 108.5 | 34.4 | 0.96 | 0.63 |
| 101 | chr7 | 7p14.2 | 115.3 | 50.7 | 118.5 | 35.6 | 1.03 | 0.7 |
| 102 | chr7 | 7p14.1 | 141.8 | 39.8 | 157 | 38.4 | 1.11 | 0.97 |
| 103 | chr7 | 7p14.1 | 117.8 | 32.5 | 74.9 | 18.5 | 0.64 | 0.57 |
| 104 | chr7 | 7p14.1 | 129.6 | 44 | 119 | 28.8 | 0.92 | 0.65 |
| 105 | chr7 | 7p14.1 | 102.3 | 54.3 | 63.8 | 29.2 | 0.62 | 0.54 |
| 106 | chr7 | 7p14.1 | 63.3 | 91.2 | 46.2 | 55.9 | 0.73 | 0.61 |
| 107 | chr7 | 7p14.1 | 134.6 | 47.6 | 191.4 | 47.2 | 1.42 | 0.99 |
| 108 | chr7 | 7p13 | 115.5 | 47.3 | 69.3 | 27.7 | 0.6 | 0.59 |
| 109 | chr7 | 7p12.3 | 114.8 | 47.5 | 78.7 | 26.8 | 0.69 | 0.57 |
| 110 | chr7 | 7p12.1 | 121 | 28.7 | 207.4 | 88.3 | 1.71 | 3.08 |
| 111 | chr7 | 7q11.23 | 109 | 53.3 | 69.8 | 27.2 | 0.64 | 0.51 |
| 112 | chr7 | 7q11.23 | 126.3 | 53 | 115.1 | 36.3 | 0.91 | 0.69 |
| 113 | chr7 | 7q21.3 | 108 | 59.2 | 83.1 | 38.1 | 0.77 | 0.64 |
| 114 | chr7 | 7q22.1 | 126.7 | 32.8 | 79.8 | 20.5 | 0.63 | 0.63 |
| 115 | chr7 | 7q22.1 | 98.6 | 77.6 | 82.5 | 52 | 0.84 | 0.67 |
| 116 | chr7 | 7q31.31 | 101.3 | 53.2 | 62.5 | 27.2 | 0.62 | 0.51 |
| 117 | chr7 | 7q31.32 | 88.7 | 75.1 | 58.1 | 39.2 | 0.65 | 0.52 |
| 118 | chr7 | 7q36.1 | 123.5 | 37.1 | 88.4 | 19 | 0.72 | 0.51 |
| 119 | chr7 | 7q36.2 | 86.2 | 67.1 | 56.2 | 37.9 | 0.65 | 0.57 |
| 120 | chr8 | 8p21.3 | 111.8 | 61 | 92.5 | 41.5 | 0.83 | 0.68 |
| 121 | chr8 | 8p21.2 | 113.3 | 73.1 | 102.9 | 58 | 0.91 | 0.79 |
| 122 | chr8 | 8p21.2 | 106.1 | 63.1 | 92.2 | 42.9 | 0.87 | 0.68 |
| 123 | chr8 | 8p21.2 | 106.8 | 61.2 | 83.6 | 38.9 | 0.78 | 0.64 |
| 124 | chr8 | 8p21.1 | 87.4 | 57.5 | 45.1 | 25.9 | 0.52 | 0.45 |
| 125 | chr8 | 8q12.1 | 128.1 | 56.2 | 133.9 | 47.6 | 1.05 | 0.85 |
| 126 | chr8 | 8q22.2 | 107.1 | 50.2 | 80.3 | 22.8 | 0.75 | 0.45 |
| 127 | chr8 | 8q22.2 | 124 | 49.4 | 115.5 | 32.6 | 0.93 | 0.66 |
| 128 | chr8 | 8q23.3 | 103.4 | 42.8 | 59.3 | 23.2 | 0.57 | 0.54 |
| 129 | chr8 | 8q23.3 | 85 | 58.8 | 46 | 31.7 | 0.54 | 0.54 |
| 130 | chr9 | 9p13.2 | 97.5 | 49.9 | 60.4 | 22.5 | 0.62 | 0.45 |
| 131 | chr9 | 9q22.31 | 126.5 | 47.1 | 105.8 | 31.5 | 0.84 | 0.67 |
| 132 | chr9 | 9q22.32 | 88.5 | 58.8 | 64.2 | 29.6 | 0.73 | 0.5 |
| 133 | chr9 | 9q22.32 | 114.6 | 57.3 | 86.7 | 33.1 | 0.76 | 0.58 |
| 134 | chr9 | 9q31.3 | 113.4 | 56.1 | 92 | 38.5 | 0.81 | 0.69 |
| 135 | chr9 | 9q33.3 | 90.1 | 63.2 | 51 | 35.9 | 0.57 | 0.57 |
| 136 | chr9 | 9q33.3 | 113.8 | 64.2 | 101.4 | 39.8 | 0.89 | 0.62 |
| 137 | chr9 | 9q34.11 | 127.4 | 50.8 | 100.5 | 34.1 | 0.79 | 0.67 |
| 138 | chr9 | 9q34.13 | 97.1 | 46.6 | 46.4 | 19.5 | 0.48 | 0.42 |
| 139 | chr10 | 10p15.1 | 78.8 | 73.6 | 49.4 | 45.9 | 0.63 | 0.62 |
| 140 | chr10 | 10p14 | 105 | 49.3 | 62.7 | 25.8 | 0.6 | 0.52 |
| 141 | chr10 | 10p14 | 110.5 | 40.5 | 60.3 | 19.2 | 0.55 | 0.47 |
| 142 | chr10 | 10p14 | 90.6 | 51.5 | 57.6 | 25.3 | 0.64 | 0.49 |
| 143 | chr10 | 10p14 | 110.5 | 36.3 | 71.7 | 20.6 | 0.65 | 0.57 |
| 144 | chr10 | 10p14 | 41.7 | 55.6 | 126.8 | 211.8 | 3.04 | 3.81 |
| 145 | chr10 | 10p14 | 88.6 | 65.1 | 52.3 | 34.9 | 0.59 | 0.54 |
| 146 | chr10 | 10p13 | 104.5 | 63.1 | 75.5 | 34.3 | 0.72 | 0.54 |
| 147 | chr10 | 10p13 | 67.9 | 73.1 | 38.3 | 35.7 | 0.56 | 0.49 |
| 148 | chr10 | 10p11.22 | 101.8 | 56.7 | 58.5 | 24.1 | 0.57 | 0.43 |
| 149 | chr10 | 10q21.3 | 117 | 51.4 | 87.2 | 28 | 0.75 | 0.55 |
| 150 | chr10 | 10q24.1 | 117 | 48.3 | 96.5 | 24.4 | 0.83 | 0.5 |
| 151 | chr10 | 10q24.2 | 123.1 | 62.2 | 120.7 | 45.6 | 0.98 | 0.73 |
| 152 | chr10 | 10q25.2 | 118.9 | 52.1 | 101.7 | 31.3 | 0.86 | 0.6 |
| 153 | chr10 | 10q25.3 | 77 | 76.9 | 46 | 44.3 | 0.6 | 0.58 |
| 154 | chr11 | 11p15.4 | 109.3 | 58.8 | 81.7 | 36.7 | 0.75 | 0.62 |
| 155 | chr11 | 11q13.3 | 103.4 | 48.2 | 57.7 | 25.6 | 0.56 | 0.53 |
| 156 | chr11 | 11q14.1 | 94.9 | 57 | 63.7 | 29.3 | 0.67 | 0.51 |
| 157 | chr11 | 11q14.2 | 91.2 | 77.1 | 83.6 | 48.1 | 0.92 | 0.62 |
| 158 | chr11 | 11q14.2 | 97.5 | 72.4 | 91.2 | 56.8 | 0.94 | 0.78 |
| 159 | chr11 | 11q21 | 98.6 | 54 | 91.3 | 50.5 | 0.93 | 0.94 |
| 160 | chr11 | 11q23.3 | 89.3 | 61.1 | 56.8 | 34.2 | 0.64 | 0.56 |
| 161 | chr11 | 11q24.2 | 104.9 | 59.5 | 91.6 | 41.7 | 0.87 | 0.7 |
| 162 | chr11 | 11q24.3 | 124.2 | 45.4 | 104.1 | 36.7 | 0.84 | 0.81 |
| 163 | chr11 | 11q25 | 119.5 | 53.6 | 115.6 | 36.6 | 0.97 | 0.68 |
| 164 | chr12 | 12p13.32 | 108 | 63.5 | 92.1 | 40.2 | 0.85 | 0.63 |
| 165 | chr12 | 12p13.31 | 132.3 | 50.9 | 156.7 | 43.2 | 1.18 | 0.85 |
| 166 | chr12 | 12p13.2 | 103.2 | 48.2 | 58.5 | 24.1 | 0.57 | 0.5 |
| 167 | chr12 | 12p13.2 | 113.5 | 42.8 | 75.1 | 24.6 | 0.66 | 0.58 |
| 168 | chr12 | 12p12.3 | 92.4 | 49.1 | 47.7 | 20.9 | 0.52 | 0.43 |
| 169 | chr12 | 12p12.1 | 90.6 | 66.3 | 53.6 | 37.6 | 0.59 | 0.57 |
| 170 | chr12 | 12q13.12 | 119.8 | 56.6 | 118.2 | 35.9 | 0.99 | 0.63 |
| 171 | chr12 | 12q13.12 | 87.8 | 75.2 | 58.9 | 50.4 | 0.67 | 0.67 |
| 172 | chr12 | 12q13.13 | 110.1 | 38.2 | 59.2 | 18.5 | 0.54 | 0.49 |
| 173 | chr12 | 12q24.21 | 120.7 | 49.8 | 88.3 | 31.7 | 0.73 | 0.64 |
| 174 | chr12 | 12q24.21 | 107.1 | 55 | 73.6 | 31.6 | 0.69 | 0.57 |
| 175 | chr12 | 12q24.31 | 108.2 | 71 | 89.2 | 59.3 | 0.82 | 0.83 |
| 176 | chr13 | 13q12.11 | 114 | 70.1 | 114.1 | 50.9 | 1 | 0.73 |
| 177 | chr13 | 13q12.2 | 85.3 | 78.7 | 62.6 | 50.9 | 0.73 | 0.65 |
| 178 | chr13 | 13q12.2 | 124.4 | 57.3 | 136.1 | 41 | 1.09 | 0.72 |
| 179 | chr13 | 13q12.3 | 111.1 | 53.8 | 82.9 | 29.6 | 0.75 | 0.55 |
| 180 | chr13 | 13q13.1 | 88.2 | 54.8 | 45.1 | 22.9 | 0.51 | 0.42 |
| 181 | chr13 | 13q13.1 | 103.4 | NA | 330.8 | 0 | 3.2 | NA |
| 182 | chr13 | 13q14.11 | 67.8 | 75.1 | 41 | 38 | 0.6 | 0.51 |
| 183 | chr13 | 13q14.11 | 76.1 | 61.2 | 38.9 | 29.5 | 0.51 | 0.48 |
| 184 | chr13 | 13q14.2 | 96.2 | 61.3 | 58.9 | 37.3 | 0.61 | 0.61 |
| 185 | chr13 | 13q14.2 | 116.9 | 49.1 | 90.8 | 28.3 | 0.78 | 0.58 |
| 186 | chr13 | 13q14.2 | 103.3 | 67.2 | 85.9 | 48 | 0.83 | 0.71 |
| 187 | chr13 | 13q14.3 | 102.7 | 60.8 | 70.9 | 35.5 | 0.69 | 0.58 |
| 188 | chr13 | 13q14.3 | 114.9 | 44.6 | 79 | 25 | 0.69 | 0.56 |
| 189 | chr13 | 13q14.3 | 70.8 | 73 | 40.6 | 35.7 | 0.57 | 0.49 |
| 190 | chr13 | 13q21.33 | 127 | 52.4 | 152.6 | 42 | 1.2 | 0.8 |
| 191 | chr13 | 13q31.2 | 93 | 51.8 | 58.7 | 24.9 | 0.63 | 0.48 |
| 192 | chr13 | 13q32.1 | 99.6 | 48.6 | 59.6 | 25 | 0.6 | 0.51 |
| 193 | chr13 | 13q32.1 | 97.9 | 52.5 | 56.9 | 24 | 0.58 | 0.46 |
| 194 | chr13 | 13q32.3 | 101.6 | 63.1 | 75.3 | 37.2 | 0.74 | 0.59 |
| 195 | chr13 | 13q32.3 | 134.6 | 40.1 | 128.2 | 27.7 | 0.95 | 0.69 |
| 196 | chr13 | 13q32.3 | 98.2 | 59.3 | 60.2 | 32.4 | 0.61 | 0.55 |
| 197 | chr13 | 13q33.1 | 92.1 | 68.1 | 62.8 | 37.1 | 0.68 | 0.55 |
| 198 | chr13 | 13q34 | 101.1 | 61.7 | 73.1 | 36.7 | 0.72 | 0.59 |
| 199 | chr13 | 13q34 | 89 | 64.9 | 55.5 | 33.3 | 0.62 | 0.51 |
| 200 | chr13 | 13q34 | 80.9 | 54.3 | 47.2 | 25.4 | 0.58 | 0.47 |
| 201 | chr13 | 13q34 | 113 | 39.2 | 79.2 | 21.5 | 0.7 | 0.55 |
| 202 | chr13 | 13q34 | 87.9 | 58.4 | 47.8 | 30.6 | 0.54 | 0.52 |
| 203 | chr14 | 14q13.3 | 77.1 | 79.4 | 50.3 | 47.4 | 0.65 | 0.6 |
| 204 | chr14 | 14q21.1 | 100.6 | 54.7 | 65.6 | 32 | 0.65 | 0.59 |
| 205 | chr14 | 14q21.1 | 93 | 66.9 | 58.2 | 33.8 | 0.63 | 0.51 |
| 206 | chr14 | 14q22.2 | 111.4 | 62.5 | 103.9 | 40.9 | 0.93 | 0.65 |
| 207 | chr14 | 14q23.1 | 98.1 | 56 | 58.5 | 25.2 | 0.6 | 0.45 |
| 208 | chr14 | 14q24.3 | 105.8 | 66 | 84.4 | 41.6 | 0.8 | 0.63 |
| 209 | chr14 | 14q24.3 | 112.1 | 65.1 | 95.3 | 44.9 | 0.85 | 0.69 |
| 210 | chr14 | 14q31.3 | 97 | 68.1 | 77.3 | 44.5 | 0.8 | 0.65 |
| 211 | chr14 | 14q32.11 | 107.4 | 69 | 120.5 | 45.9 | 1.12 | 0.67 |
| 212 | chr14 | 14q32.12 | 90.5 | 69.9 | 51.2 | 42.4 | 0.57 | 0.61 |
| 213 | chr14 | 14q32.12 | 111.3 | 52.8 | 82.8 | 27.9 | 0.74 | 0.53 |
| 214 | chr15 | 15q14 | 95.8 | 54 | 55.5 | 28.7 | 0.58 | 0.53 |
| 215 | chr15 | 15q22.31 | 76 | 62.5 | 36.7 | 33 | 0.48 | 0.53 |
| 216 | chr15 | 15q22.33 | 78.4 | 66.5 | 37.7 | 32.9 | 0.48 | 0.49 |
| 217 | chr15 | 15q22.33 | 101.2 | 46.2 | 54 | 20.9 | 0.53 | 0.45 |
| 218 | chr15 | 15q23 | 91.6 | 60.1 | 52.5 | 37.7 | 0.57 | 0.63 |
| 219 | chr15 | 15q23 | 74.7 | 80.5 | 47.1 | 45.6 | 0.63 | 0.57 |
| 220 | chr15 | 15q24.1 | 107.3 | 46 | 66.6 | 27.2 | 0.62 | 0.59 |
| 221 | chr15 | 15q26.1 | 115.8 | 53.2 | 90.4 | 34.3 | 0.78 | 0.64 |
| 222 | chr15 | 15q26.1 | 110.7 | 48.8 | 77.2 | 28.8 | 0.7 | 0.59 |
| 223 | chr16 | 16p13.13 | 100.9 | 64.7 | 84 | 42.2 | 0.83 | 0.65 |
| 224 | chr16 | 16p13.13 | 88.6 | 68 | 66 | 42.3 | 0.75 | 0.62 |
| 225 | chr16 | 16p12.1 | 103 | 38.8 | 53.9 | 12.5 | 0.52 | 0.32 |
| 226 | chr16 | 16p11.2 | 98.5 | 60.8 | 65.2 | 35.7 | 0.66 | 0.59 |
| 227 | chr16 | 16q12.1 | 120.2 | 40.8 | 74.4 | 24.3 | 0.62 | 0.6 |
| 228 | chr16 | 16q12.1 | 114.1 | 62.6 | 124.1 | 44.7 | 1.09 | 0.71 |
| 229 | chr16 | 16q12.2 | 94.8 | 42.9 | 50.8 | 15.4 | 0.54 | 0.36 |
| 230 | chr17 | 17p13.3 | 101.9 | 62.7 | 82.6 | 39.2 | 0.81 | 0.62 |
| 231 | chr17 | 17p13.1 | 90.9 | 57.9 | 54.9 | 31.1 | 0.6 | 0.54 |
| 232 | chr17 | 17p11.2 | 112.6 | 72.3 | 109.2 | 73.6 | 0.97 | 1.02 |
| 233 | chr17 | 17q11.2 | 105.2 | 44.1 | 57.5 | 16.8 | 0.55 | 0.38 |
| 234 | chr17 | 17q12 | 90.4 | 44.7 | 42.6 | 20.3 | 0.47 | 0.45 |
| 235 | chr17 | 17q23.3 | 94.9 | 59 | 57.5 | 30.6 | 0.61 | 0.52 |
| 236 | chr17 | 17q24.2 | 14.8 | 91.8 | 79.6 | 238.8 | 5.39 | 2.6 |
| 237 | chr17 | 17q24.2 | 98.9 | 62.4 | 68.6 | 34.6 | 0.69 | 0.55 |
| 238 | chr17 | 17q24.2 | 88.3 | 58.3 | 52.7 | 27.7 | 0.6 | 0.48 |
| 239 | chr17 | 17q24.3 | 120.2 | 45.9 | 85.9 | 32.8 | 0.71 | 0.71 |
| 240 | chr18 | 18p11.31 | 104.6 | 50.5 | 61.8 | 23.7 | 0.59 | 0.47 |
| 241 | chr18 | 18p11.31 | 89.4 | 56.9 | 48.2 | 28.4 | 0.54 | 0.5 |
| 242 | chr18 | 18p11.31 | 144.4 | 30.2 | 128.1 | 20.1 | 0.89 | 0.66 |
| 243 | chr18 | 18p11.31 | NA | 107.1 | 0 | 386.3 | NA | 3.61 |
| 244 | chr18 | 18p11.22 | 101.9 | 56.4 | 76.9 | 26.3 | 0.75 | 0.47 |
| 245 | chr18 | 18p11.22 | 104.8 | 67.3 | 79.9 | 44 | 0.76 | 0.65 |
| 246 | chr18 | 18p11.22 | 97.9 | 39.8 | 48.3 | 19.3 | 0.49 | 0.48 |
| 247 | chr18 | 18p11.22 | 126.9 | 54.2 | 114.4 | 31.4 | 0.9 | 0.58 |
| 248 | chr18 | 18q11.2 | 111.5 | 79.1 | 99.9 | 67.4 | 0.9 | 0.85 |
| 249 | chr18 | 18q11.2 | 139.2 | 52 | 184.5 | 43.1 | 1.33 | 0.83 |
| 250 | chr18 | 18q11.2 | 96.5 | 60.3 | 60.1 | 29.1 | 0.62 | 0.48 |
| 251 | chr18 | 18q12.1 | 83.7 | 82.4 | 54.6 | 49.6 | 0.65 | 0.6 |
| 252 | chr18 | 18q12.1 | 72.5 | 71.6 | 38.8 | 36.1 | 0.53 | 0.5 |
| 253 | chr18 | 18q12.1 | 122.6 | 61.2 | 109.9 | 38.8 | 0.9 | 0.63 |
| 254 | chr18 | 18q12.2 | 124 | 60.8 | 115.7 | 37.1 | 0.93 | 0.61 |
| 255 | chr18 | 18q12.3 | 125 | 35.7 | 309.4 | 87.3 | 2.47 | 2.45 |
| 256 | chr18 | 18q21.1 | 87.7 | 69 | 59 | 38.4 | 0.67 | 0.56 |
| 257 | chr18 | 18q21.1 | 137.7 | 42.7 | 96.2 | 24.1 | 0.7 | 0.56 |
| 258 | chr18 | 18q21.1 | 121.8 | 61.4 | 105.7 | 35.5 | 0.87 | 0.58 |
| 259 | chr18 | 18q21.2 | 121.6 | 51.5 | 108.2 | 31.2 | 0.89 | 0.6 |
| 260 | chr18 | 18q21.2 | 93.9 | 54.1 | 60.2 | 23.8 | 0.64 | 0.44 |
| 261 | chr18 | 18q21.2 | 90.8 | 55.5 | 50.8 | 21.2 | 0.56 | 0.38 |
| 262 | chr18 | 18q21.31 | 85.1 | 54.6 | 45.9 | 20.4 | 0.54 | 0.37 |
| 263 | chr18 | 18q21.31 | 140 | 46.2 | 132.2 | 28.6 | 0.94 | 0.62 |
| 264 | chr18 | 18q21.32 | 104.3 | 50.3 | 63.3 | 24.1 | 0.61 | 0.48 |
| 265 | chr18 | 18q21.32 | 123.5 | 63.1 | 97.8 | 39.8 | 0.79 | 0.63 |
| 266 | chr18 | 18q21.32 | 104.7 | 82.5 | 81.9 | 47.9 | 0.78 | 0.58 |
| 267 | chr18 | 18q21.33 | 106 | 62.5 | 80.3 | 38.7 | 0.76 | 0.62 |
| 268 | chr18 | 18q21.33 | 114.6 | 67.1 | 119.7 | 48.8 | 1.04 | 0.73 |
| 269 | chr18 | 18q21.33 | 100.4 | 44.4 | 49.8 | 20.2 | 0.5 | 0.46 |
| 270 | chr18 | 18q22.3 | 108 | 51.7 | 76.1 | 26.4 | 0.71 | 0.51 |
| 271 | chr19 | 18p13.12 | 97.2 | 79.7 | 75.2 | 54.3 | 0.77 | 0.68 |
| 272 | chr19 | 19q12 | 115.1 | 36.9 | 60 | 19.8 | 0.52 | 0.54 |
| 273 | chr19 | 19q13.12 | 83.2 | 63.1 | 54.1 | 28.1 | 0.65 | 0.45 |
| 274 | chr19 | 19q13.13 | 68.2 | 75.9 | 33.9 | 39.2 | 0.5 | 0.52 |
| 275 | chr19 | 19q13.13 | 100.8 | 70.3 | 81 | 39.8 | 0.8 | 0.57 |
| 276 | chr19 | 19q13.2 | 127.5 | 49.2 | 126.2 | 33.7 | 0.99 | 0.69 |
| 277 | chr20 | 20p11.23 | 102.9 | 45.1 | 62.7 | 22.3 | 0.61 | 0.49 |
| 278 | chr20 | 20p11.23 | 100.1 | 44.7 | 59.7 | 20.5 | 0.6 | 0.46 |
| 279 | chr20 | 20p11.22 | 110.9 | 41.5 | 63.5 | 20.3 | 0.57 | 0.49 |
| 280 | chr20 | 20p11.22 | 99.1 | 64.2 | 73.9 | 38.1 | 0.75 | 0.59 |
| 281 | chr20 | 20q11.23 | 116 | 51.4 | 92.3 | 32.8 | 0.8 | 0.64 |
| 282 | chr20 | 20q13.2 | 111.5 | 54.1 | 86.8 | 33.9 | 0.78 | 0.63 |
| 283 | chr20 | 20q13.31 | 107.6 | 54.8 | 76.8 | 36.3 | 0.71 | 0.66 |
| 284 | chr20 | 20q13.31 | 106.5 | 59.2 | 79.8 | 31.9 | 0.75 | 0.54 |
| 285 | chr20 | 20q13.32 | 110.8 | 44.9 | 68.1 | 26.6 | 0.61 | 0.59 |
| 286 | chr21 | 21q11.2 | 142.5 | 32.7 | 154.5 | 28 | 1.08 | 0.86 |
| 287 | chr21 | 21q21.1 | 101.1 | 41.1 | 57 | 17.2 | 0.56 | 0.42 |
| 288 | chr21 | 21q21.1 | 103.6 | 45.3 | 52.6 | 20.6 | 0.51 | 0.46 |
| 289 | chr21 | 21q21.1 | 90.9 | 57.7 | 46.8 | 27.2 | 0.51 | 0.47 |
| 290 | chr21 | 21q21.1 | 95.1 | 51.3 | 53.4 | 23.3 | 0.56 | 0.45 |
| 291 | chr21 | 21q21.3 | 91.5 | 52.7 | 53.8 | 27.8 | 0.59 | 0.53 |
| 292 | chr21 | 21q21.3 | 120.6 | 64.3 | 130.6 | 43.4 | 1.08 | 0.67 |
| 293 | chr21 | 21q21.3 | 78.7 | 65.4 | 48.9 | 31.4 | 0.62 | 0.48 |
| 294 | chr21 | 21q21.3 | 78.1 | 63.2 | 41.9 | 30.8 | 0.54 | 0.49 |
| 295 | chr21 | 21q21.3 | 93.7 | 64.7 | 62.3 | 35.2 | 0.66 | 0.54 |
| 296 | chr21 | 21q21.3 | 100.1 | 52.5 | 62.6 | 23.9 | 0.63 | 0.45 |
| 297 | chr21 | 21q21.3 | 82.8 | 22.2 | 264.7 | 115.5 | 3.2 | 5.2 |
| 298 | chr21 | 21q22.11 | 100.4 | 49.6 | 58.5 | 24.6 | 0.58 | 0.5 |
| 299 | chr21 | 21q22.11 | 73.6 | 104.4 | 56 | 61.8 | 0.76 | 0.59 |
| 300 | chr21 | 21q22.11 | 123.9 | 43.4 | 79.4 | 22.2 | 0.64 | 0.51 |
| 301 | chr21 | 21q22.11 | 98.6 | 57 | 63 | 32.5 | 0.64 | 0.57 |
| 302 | chr21 | 21q22.12 | 91.1 | 62.8 | 56.1 | 34.3 | 0.62 | 0.55 |
| 303 | chr21 | 21q22.13 | 101.8 | 49.7 | 56.6 | 21.9 | 0.56 | 0.44 |
| 304 | chr21 | 21q22.13 | 122.1 | 34.3 | 77.5 | 17.3 | 0.63 | 0.5 |
| 305 | chr21 | 21q22.13 | 122.4 | 37.1 | 87.6 | 20.2 | 0.72 | 0.54 |
| 306 | chr21 | 21q22.13 | 104.7 | 50.6 | 65.5 | 27.7 | 0.63 | 0.55 |
| 307 | chr21 | 21q22.2 | 90.1 | 49.8 | 59 | 23.1 | 0.65 | 0.46 |
| 308 | chr21 | 21q22.2 | 117 | 45.2 | 103.8 | 30 | 0.89 | 0.66 |
| 309 | chr21 | 21q22.2 | 214.3 | 7.9 | 696.7 | 41.2 | 3.25 | 5.2 |
| 310 | chr21 | 21q22.2 | 90.8 | 59 | 60.2 | 26 | 0.66 | 0.44 |
| 311 | chr21 | 21q22.2 | 109 | 48.1 | 73.8 | 22.4 | 0.68 | 0.47 |
| 312 | chr21 | 21q22.2 | 147.5 | 43.5 | 130.7 | 27.2 | 0.89 | 0.62 |
| 313 | chr21 | 21q22.2 | 156.1 | 35.7 | 180.8 | 24.6 | 1.16 | 0.69 |
| 314 | chr21 | 21q22.2 | 134.3 | 45 | 121.6 | 24.6 | 0.91 | 0.55 |
| 315 | chr21 | 21q22.3 | 118.4 | 53.7 | 92.2 | 33 | 0.78 | 0.62 |
| 316 | chr21 | 21q22.3 | 108.6 | 60.1 | 75.1 | 37.9 | 0.69 | 0.63 |
| 317 | chr21 | 21q22.3 | 128.2 | 55.9 | 109.6 | 35.3 | 0.85 | 0.63 |
| 318 | chr21 | 21q22.3 | 116.8 | 51.5 | 88.9 | 31.1 | 0.76 | 0.6 |
| 319 | chr21 | 21q22.3 | 131.2 | 43.1 | 101.5 | 25.8 | 0.77 | 0.6 |
| 320 | chr21 | 21q22.3 | 132.5 | 51.2 | 119.5 | 34.6 | 0.9 | 0.68 |
| 321 | chr21 | 21q22.3 | 128.8 | 46 | 92.8 | 27.6 | 0.72 | 0.6 |
| 322 | chr21 | 21q22.3 | 151 | 53.1 | 135.2 | 41.7 | 0.9 | 0.79 |
| 323 | chr21 | 21q22.3 | 172.3 | 42.5 | 243.1 | 30.4 | 1.41 | 0.72 |
| 324 | chr21 | 21q22.3 | 212.2 | 17.5 | 312.5 | 23.9 | 1.47 | 1.37 |
| 325 | chr21 | 21q22.3 | 134.5 | 46.3 | 127.1 | 26 | 0.95 | 0.56 |
| 326 | chr22 | 22q12.2 | 87 | 52 | 43.3 | 23.8 | 0.5 | 0.46 |
| 327 | chr22 | 22q13.1 | 125.1 | 33 | 83.9 | 18.4 | 0.67 | 0.56 |
| 328 | chr22 | 22q13.1 | 112.7 | 39.3 | 76.7 | 20.3 | 0.68 | 0.52 |
| 329 | chr22 | 22q13.2 | 105.4 | 66.5 | 86.1 | 46.1 | 0.82 | 0.69 |
| 330 | chr22 | 22q13.2 | 94.9 | 70.9 | 75.8 | 39.7 | 0.8 | 0.56 |
| 331 | chr22 | 22q13.2 | 101.8 | 54.8 | 68.1 | 27.3 | 0.67 | 0.5 |
|  |  |  | **Mean Average Enrichment** | |  |  | **Mean CV** | |
|  |  |  | **108.21** | **55.60** |  |  | **0.85** | **0.69** |

CV, Coefficient of variation; CVS, Chorionic Villus Sampling; PL, non-pregnant female plasma samples
