# Supplementary material for: MeDIP combined with in-solution targeted enrichment followed by NGS: Inter-individual methylation variability of fetal-specific biomarkers and their implementation in a proof of concept study for NIPT
Source: PLoS One. 2018 Jun 11;13(6):e0199010. doi: 10.1371/journal.pone.0199010 (PMC5995407; doi:10.1371/journal.pone.0199010)
Supplement: S2 Table — (DOCX) [file pone.0199010.s003.docx]

| **DMR** | **Chr** | | **Location** | | **Mean**  **CVS** | **Mean**  **PL** | **SD CVS** | **SD PL** | | **pvalue** | | **CV CVS** | **CV PL** |
| --- | --- | --- | --- | --- | --- | --- | --- | --- | --- | --- | --- | --- | --- |
| 1 | chr1 | | p36.23 | | 114.86 | 43.3 | 103.24 | 31.38 | | 0.0246 | | 0.8988 | 0.7247 |
| 2 | chr1 | | p36.22 | | 113.01 | 45.28 | 84.82 | 32.72 | | 0.0105 | | 0.7505 | 0.7225 |
| 3 | chr1 | | p36.13 | | 110.97 | 47.48 | 86.24 | 26.28 | | 0.0165 | | 0.7771 | 0.5535 |
| 4 | chr1 | | p36.11 | | 106.12 | 52.69 | 76.99 | 28.43 | | 0.0241 | | 0.7255 | 0.5396 |
| 5 | chr1 | | p34.2 | | 115.55 | 42.56 | 84.2 | 26.26 | | 0.0036 | | 0.7287 | 0.617 |
| 6 | chr1 | | p21.3 | | 105.22 | 53.66 | 56.6 | 25.02 | | 0.0028 | | 0.5379 | 0.4663 |
| 7 | chr1 | | p21.2 | | 117.14 | 40.85 | 60.85 | 17.86 | | 1,90E+09 | | 0.5194 | 0.4371 |
| 8 | chr1 | | q31.3 | | 115.97 | 42.1 | 65.12 | 18.89 | | 0.0001 | | 0.5614 | 0.4486 |
| 9 | chr1 | | q41 | | 119.01 | 38.84 | 87.01 | 20.46 | | 0.0017 | | 0.7311 | 0.5266 |
| 10 | chr1 | | q41 | | 108.96 | 49.64 | 75.89 | 32.42 | | 0.0143 | | 0.6965 | 0.653 |
| 11 | chr2 | | p21 | | 115.72 | 42.38 | 127.44 | 35.61 | | 0.0375 | | 1.1013 | 0.8401 |
| 12 | chr2 | | p13.3 | | 120.89 | 36.83 | 89.25 | 22.56 | | 0.0013 | | 0.7382 | 0.6127 |
| 13 | chr2 | | q32.3 | | 111.8 | 46.58 | 73.98 | 27.88 | | 0.0034 | | 0.6616 | 0.5984 |
| 14 | chr2 | | q37.1 | | 121.64 | 36.01 | 76.39 | 18.88 | | 0.0001 | | 0.6279 | 0.5242 |
| 15 | chr3 | | p14.3 | | 117.43 | 40.54 | 75.14 | 21.9 | | 0.0005 | | 0.6399 | 0.5403 |
| 16 | chr3 | | q26.32 | | 113.05 | 45.24 | 74.52 | 17.26 | | 0.002 | | 0.6592 | 0.3814 |
| 17 | chr3 | | q27.1 | | 116.5 | 41.54 | 113.73 | 26.96 | | 0.0241 | | 0.9762 | 0.6489 |
| 18 | chr4 | | p14 | | 110.38 | 48.11 | 71.14 | 27.96 | | 0.0038 | | 0.6444 | 0.5811 |
| 19 | chr4 | | q26 | | 101.17 | 58 | 74.48 | 29.34 | | 0.0375 | | 0.7361 | 0.5059 |
| 20 | chr4 | | q31.1 | | 102.81 | 56.24 | 78.11 | 31.94 | | 0.0375 | | 0.7597 | 0.5678 |
| 21 | chr5 | | p13.1 | | 119.28 | 38.56 | 84.7 | 23.04 | | 0.0012 | | 0.71 | 0.5975 |
| 22 | chr6 | | p22.3 | | 117.62 | 40.33 | 104.69 | 28.05 | | 0.0159 | | 0.8901 | 0.6953 |
| 23 | chr6 | | q21 | | 121.41 | 36.26 | 127.59 | 29.43 | | 0.0241 | | 1.0509 | 0.8116 |
| 24 | chr6 | | q21 | | 119.06 | 38.78 | 96.09 | 25.81 | | 0.0049 | | 0.807 | 0.6653 |
| 25 | chr6 | | q23.3 | | 111.83 | 46.55 | 109.43 | 30.74 | | 0.033 | | 0.9785 | 0.6603 |
| 26 | chr7 | | p15.3 | | 110.6 | 47.87 | 68.59 | 26.4 | | 0.0024 | | 0.6201 | 0.5514 |
| 27 | chr7 | | p14.2 | | 110.13 | 48.38 | 113.13 | 34.01 | | 0.0375 | | 1.0272 | 0.7029 |
| 28 | chr7 | | p14.1 | | 123.04 | 34.52 | 136.22 | 33.35 | | 0.0244 | | 1.1072 | 0.9663 |
| 29 | chr7 | | p14.1 | | 123.5 | 34.02 | 78.54 | 19.37 | | 9,47E+08 | | 0.6359 | 0.5692 |
| 30 | chr7 | | p14.1 | | 117.88 | 40.05 | 108.26 | 26.18 | | 0.0183 | | 0.9183 | 0.6536 |
| 31 | chr7 | | p13 | | 112.33 | 46.02 | 67.36 | 26.96 | | 0.001 | | 0.5996 | 0.5859 |
| 32 | chr7 | | p12.3 | | 112.01 | 46.35 | 76.76 | 26.2 | | 0.0043 | | 0.6853 | 0.5651 |
| 33 | chr7 | | q11.23 | | 106.62 | 52.15 | 68.3 | 26.57 | | 0.010374 | | 0.6405 | 0.509 |
| 34 | chr7 | | q11.23 | | 111.58 | 46.82 | 101.66 | 32.08 | | 0.0253 | | 0.911 | 0.6852 |
| 35 | chr7 | | q22.1 | | 125.05 | 32.35 | 78.77 | 20.28 | | 5,59E+09 | | 0.6299 | 0.6269 |
| 36 | chr7 | | q36.1 | | 121.26 | 36.42 | 86.73 | 18.62 | | 0.0008 | | 0.7152 | 0.5112 |
| 37 | chr8 | | p21.2 | | 99.85 | 59.42 | 86.81 | 40.38 | | 0.0476 | | 0.8694 | 0.6795 |
| 38 | chr8 | | q23.3 | | 112 | 46.37 | 64.24 | 25.13 | | 0.0006 | | 0.5736 | 0.542 |
| 39 | chr9 | | q22.31 | | 115.21 | 42.92 | 96.4 | 28.72 | | 0.0159 | | 0.8367 | 0.6691 |
| 40 | chr9 | | q33.3 | | 101.71 | 57.42 | 90.6 | 35.58 | | 0.0476 | | 0.8907 | 0.6196 |
| 41 | chr10 | | p14 | | 115.72 | 42.38 | 63.23 | 20.06 | | 7,59E+09 | | 0.5463 | 0.4734 |
| 42 | chr10 | | q21.3 | | 110.13 | 48.38 | 82.12 | 26.4 | | 0.0159 | | 0.7456 | 0.5457 |
| 43 | chr10 | | q24.1 | | 112.06 | 46.3 | 92.47 | 23.32 | | 0.0184 | | 0.8251 | 0.5036 |
| 44 | chr10 | | q24.2 | | 105.53 | 53.32 | 103.49 | 39.14 | | 0.0476 | | 0.9806 | 0.7341 |
| 45 | chr10 | | q25.2 | | 110.21 | 48.29 | 94.24 | 29 | | 0.0244 | | 0.855 | 0.6003 |
| 46 | chr12 | | q13.13 | | 117.3 | 40.67 | 63.08 | 19.76 | | 3,46E+09 | | 0.5377 | 0.4859 |
| 47 | chr12 | | q24.21 | | 104.94 | 53.95 | 72.15 | 31.02 | | 0.0241 | | 0.6874 | 0.5749 |
| 48 | chr13 | | q14.2 | | 111.56 | 46.84 | 86.69 | 27.03 | | 0.0159 | | 0.777 | 0.577 |
| 49 | chr13 | | q14.3 | | 114.02 | 44.2 | 78.34 | 24.82 | | 0.0027 | | 0.687 | 0.5615 |
| 50 | chr13 | | q34 | | 117.32 | 40.66 | 82.26 | 22.28 | | 0.0016 | | 0.7011 | 0.5479 |
| 51 | chr14 | | q32.12 | | 107.64 | 51.05 | 80.15 | 26.98 | | 0.0222 | | 0.7445 | 0.5283 |
| 52 | chr15 | | q22.33 | | 108.89 | 49.71 | 58.1 | 22.5 | | 0.0006 | | 0.5335 | 0.4525 |
| 53 | chr15 | | q23 | | 110.91 | 47.54 | 68.82 | 28.15 | | 0.0024 | | 0.6204 | 0.592 |
| 54 | chr15 | | q24.1 | | 108.65 | 49.97 | 84.83 | 32.17 | | 0.0241 | | 0.7808 | 0.6436 |
| 55 | chr15 | | q26.1 | | 110 | 48.51 | 76.73 | 28.67 | | 0.01 | | 0.6975 | 0.5908 |
| 56 | chr16 | | p13.13 | | 114.86 | 43.29 | 60.12 | 13.93 | | 4,61E+09 | | 0.5234 | 0.3218 |
| 57 | chr16 | | q12.1 | | 109.14 | 49.44 | 58.49 | 17.71 | | 0.0005 | | 0.5358 | 0.3581 |
| 58 | chr17 | | p11.2 | | 111.65 | 46.74 | 61.05 | 17.8 | | 0.0002 | | 0.5467 | 0.3807 |
| 59 | chr18 | | p11.31 | | 129.87 | 27.17 | 115.22 | 18.06 | | 0.0024 | | 0.8871 | 0.6644 |
| 60 | chr18 | | p11.22 | | 112.56 | 45.77 | 55.45 | 22.15 | | 4,65E+09 | | 0.4926 | 0.4839 |
| 61 | chr18 | | q21.1 | | 111.27 | 47.15 | 99.02 | 28.51 | | 0.0244 | | 0.8899 | 0.6046 |
| 62 | chr18 | | q21.33 | | 109.93 | 48.6 | 54.57 | 22.15 | | 0.0001 | | 0.4963 | 0.4557 |
| 63 | chr19 | | p13.12 | | 119.51 | 38.3 | 62.35 | 20.6 | | 9,53E+07 | | 0.5216 | 0.5377 |
| 64 | chr19 | | q13.13 | | 114.18 | 44.03 | 113.08 | 30.16 | | 0.0263 | | 0.9903 | 0.685 |
| 65 | chr20 | | p11.23 | | 115.11 | 43.03 | 65.94 | 21.1 | | 0.0001 | | 0.5728 | 0.4902 |
| 66 | chr20 | | q13.31 | | 112.69 | 45.63 | 69.29 | 27.08 | | 0.0012 | | 0.6148 | 0.5934 |
| 67 | chr20 | | q13.32 | | 127.85 | 29.35 | 138.58 | 25.14 | | 0.0183 | | 1.0839 | 0.8567 |
| 68 | chr21 | | q21.1 | | 110.25 | 48.24 | 55.98 | 21.97 | | 0.0001 | | 0.5076 | 0.4553 |
| 69 | chr21 | | q22.11 | | 117 | 41 | 74.95 | 20.93 | | 0.0005 | | 0.6406 | 0.5104 |
| 70 | chr21 | | q22.13 | | 123.01 | 34.55 | 78.1 | 17.42 | | 0.0001 | | 0.6349 | 0.5041 |
| 71 | chr21 | | q22.13 | | 121 | 36.7 | 86.57 | 20 | | 0.0008 | | 0.7154 | 0.5448 |
| 72 | chr21 | | q22.13 | | 102.44 | 56.64 | 67.09 | 26.23 | | 0.0241 | | 0.6548 | 0.4631 |
| 73 | chr21 | | q22.2 | | 114.11 | 44.1 | 101.22 | 29.28 | | 0.0236 | | 0.887 | 0.6638 |
| 74 | chr21 | | q22.2 | | 118.3 | 39.6 | 107.08 | 21.63 | | 0.0159 | | 0.9051 | 0.5462 |
| 75 | chr21 | | q22.3 | | 118.85 | 39.01 | 91.92 | 23.38 | | 0.0033 | | 0.7734 | 0.5993 |
| 76 | chr21 | | q22.3 | | 116.46 | 41.59 | 83.96 | 24.97 | | 0.0027 | | 0.7209 | 0.6004 |
| 77 | chr21 | | q22.3 | | 116.88 | 41.13 | 104.67 | 32.29 | | 0.0183 | | 0.8955 | 0.7851 |
| 78 | chr22 | | q13.1 | | 124.58 | 32.85 | 83.49 | 18.32 | | 0.0001 | | 0.6701 | 0.5577 |
|  | |  | |  | **Mean Average Enrichment** | |  | |  | |  | Mean Coefficient of Variation | |
|  |  | |  | | **113.93** | **44.3** |  |  | |  | | **0.7365** | **0.5824** |

Chr, Chromosome; CV, Coefficient of variation; CVS, Chorionic Villus Sampling; PL, non-pregnant female plasma sample
