# Supplementary material for: MeDIP combined with in-solution targeted enrichment followed by NGS: Inter-individual methylation variability of fetal-specific biomarkers and their implementation in a proof of concept study for NIPT
Source: PLoS One. 2018 Jun 11;13(6):e0199010. doi: 10.1371/journal.pone.0199010 (PMC5995407; doi:10.1371/journal.pone.0199010)
Supplement: S3 Table — (DOCX) [file pone.0199010.s004.docx]

| Cases | %Fetal  Fraction |
| --- | --- |
|  |  |
| Euploid_1 | 16% |
| Euploid_2 | 13.5% |
| Euploid_3 | 12% |
| Euploid_4 | 9.6% |
| Euploid_5 | 9.5% |
| Euploid_6 | 9% |
| Euploid_7 | 8.6% |
| Euploid_8 | 8.99% |
| Euploid_9 | 6.5% |
| Euploid_10 | 15% |
| Euploid_11 | 9.8% |
| Euploid_12 | 9.2% |
| Euploid_13 | 8.8% |
| Euploid_14 | 8.9% |
| Euploid_15 | 5.85% |
| Euploid_16 | 6.9% |
| Euploid_17 | 9.7% |
| Euploid_18 | 6.5% |
| Euploid_19 | 6.9% |
| Euploid_20 | 5.13% |
| Euploid_21 | 10.5% |
| Euploid_22 | 11.7% |
| Euploid_23 | 5.55% |
| Euploid_24 | 9.6% |
| Euploid_25 | 6.7% |
| Euploid_26 | 10.5% |
| Euploid_27 | 6.99% |
| Euploid_28 | 8.47% |
| Euploid_29 | 10.84% |
| Euploid_30 | 4.55% |
| Euploid_31 | 11.23% |
| Euploid_32 | 11.6% |
| Euploid_33 | 7.89% |
| Euploid_34 | 6.52% |
| Euploid_35 | 6.79% |
| Euploid_36 | 9% |
| Euploid_37 | 8.2% |
| Euploid_38 | 7.9% |
| Trisomy-13 | 5.82% |
| Trisomy-18 | 6.1% |
| Trisomy-21_1 | 9.14% |
| Trisomy-21_2 | 8% |
| Trisomy-21_3 | 9% |
| Trisomy-21_4 | 5.96% |
